# Multi-animal pose estimation, identification and tracking with DeepLabCut

**DOI:** 10.1038/s41592-022-01443-0

**Published:** 2022-04-12

**Authors:** Jessy Lauer, Mu Zhou, Shaokai Ye, William Menegas, Steffen Schneider, Tanmay Nath, Mohammed Mostafizur Rahman, Valentina Di Santo, Daniel Soberanes, Guoping Feng, Venkatesh N. Murthy, George Lauder, Catherine Dulac, Mackenzie Weygandt Mathis, Alexander Mathis

**Affiliations:** 1 Brain Mind Institute, School of Life Sciences, Swiss Federal Institute of Technology (EPFL), Lausanne, Switzerland; 2 Rowland Institute at Harvard, Harvard University, Cambridge, MA USA; 3 Department of Brain and Cognitive Sciences and McGovern Institute for Brain Research, Massachusetts Institute of Technology, Cambridge, MA USA; 4 Department for Molecular Biology and Center for Brain Science, Harvard University, Cambridge, MA USA; 5 Howard Hughes Medical Institute (HHMI), Chevy Chase, MD USA; 6 Department of Organismic and Evolutionary Biology, Harvard University, Cambridge, MA USA; 7 Department of Zoology, Stockholm University, Stockholm, Sweden

**Keywords:** Machine learning, Computational neuroscience, Zoology, Behavioural methods

## Abstract

Estimating the pose of multiple animals is a challenging computer vision problem: frequent interactions cause occlusions and complicate the association of detected keypoints to the correct individuals, as well as having highly similar looking animals that interact more closely than in typical multi-human scenarios. To take up this challenge, we build on DeepLabCut, an open-source pose estimation toolbox, and provide high-performance animal assembly and tracking—features required for multi-animal scenarios. Furthermore, we integrate the ability to predict an animal’s identity to assist tracking (in case of occlusions). We illustrate the power of this framework with four datasets varying in complexity, which we release to serve as a benchmark for future algorithm development.

## Abstract (web summary)

DeepLabCut is extended to enable multi-animal pose estimation, animal identification and tracking, thereby enabling the analysis of social behaviors.

## Main

Advances in sensor and transmitter technology, data mining and computational analysis herald a golden age of animal tracking across the globe^1^. Computer vision is a crucial tool for identifying, counting, as well as annotating animal behavior^2–4^. For the computational analysis of fine-grained behavior, pose estimation is often a crucial step and deep-learning based tools have quickly affected neuroscience, ethology and medicine^5–8^.

Many experiments in biology—from parenting mice to fish schooling—require measuring interactions among multiple individuals. Multi-animal pose estimation raises several challenges that can leverage advances in machine vision research, and yet others that need new solutions. In general, the process requires three steps: pose estimation (that is, keypoint localization), assembly (that is, the task of grouping keypoints into distinct animals) and tracking. Each step presents different challenges.

To make pose estimation robust to interacting and occluded animals, one should annotate frames with closely interacting animals. To associate detected keypoints to particular individuals (assembly) several solutions have been proposed, such as part affinity fields (PAFs)^9^, associative embeddings^10,11^, transformers^12^ and other mechanisms^13,14^. Tracking animals between frames can be difficult because of appearance similarity, nonstationary behaviors and possible occlusions. Building on human pose estimation research, some packages for multi-animal pose estimation have emerged^15–17^. Here, we developed top-performing network architectures, a data-driven assembly method, engineered tailored tracking methods and compared the current state-of-the-art networks on COCO (common objects in context)^18^ on four animal datasets.

Specifically, we expanded DeepLabCut^19–21^, an open-source toolbox for animal pose estimation. Our contributions are as follows:

1. Four datasets of varying difficulty for benchmarking multi-animal pose estimation networks.
2. Multi-task architecture that predicts multiple conditional random fields and therefore can predict keypoints, limbs, as well as animal identity.
3. A data-driven method for animal assembly that finds the optimal skeleton without user input, and that is state of the art (compared to top-models from COCO, a standard computer vision benchmark).
4. A module that casts tracking as a network flow optimization problem, which aims to find globally optimal solutions.
5. Unsupervised animal ID tracking: we can predict the identity of animals and reidentify them; this is particularly useful to link animals across time when temporally based tracking fails (due to intermittent occlusions).
6. Graphical user interfaces (GUIs) for keypoint annotation, refinement and semiautomatic trajectory verification.

## Results

**Extended Data Fig. 1:**
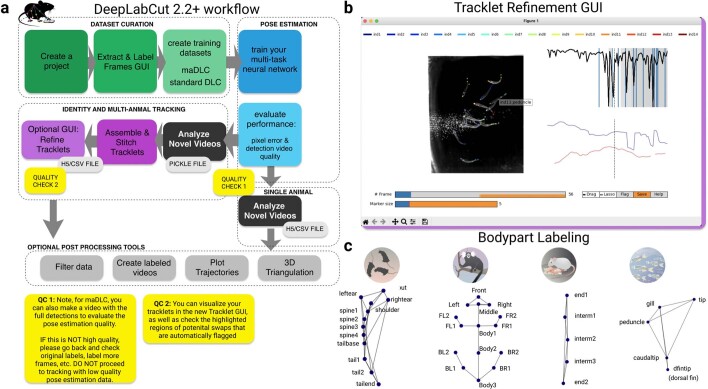
DeepLabCut 2.2 workflow. (a) Multi-animal DeepLabCut2.2+ workflow. (b) An example screenshot of the Refine Tracklet GUI. We show the ellipse similarity score (black line), hand-noted GT switches in ID (blue), and additional frames in orange where the selected keypoint requires further examination. (c) Body part keypoint diagrams with names on the animal skeletons (see also Extended Data Figure 2).

Multi-animal pose estimation can be cast as a data assignment problem in the spatial and temporal domains. To tackle the generic multi-animal pose-tracking scenario, we designed a practical, almost entirely data-driven solution that breaks down the larger goal into the smaller subtasks of: keypoint estimation, animal assembly (spatially grouping keypoints into individuals), local (temporal) tracking and global ‘tracklet’ stitching (Extended Data Fig. 1). We evaluate our pipeline on four new datasets that we release with this paper as a benchmark at https://benchmark.deeplabcut.org/.

### Four diverse multi-animal datasets

**Fig. 1:**
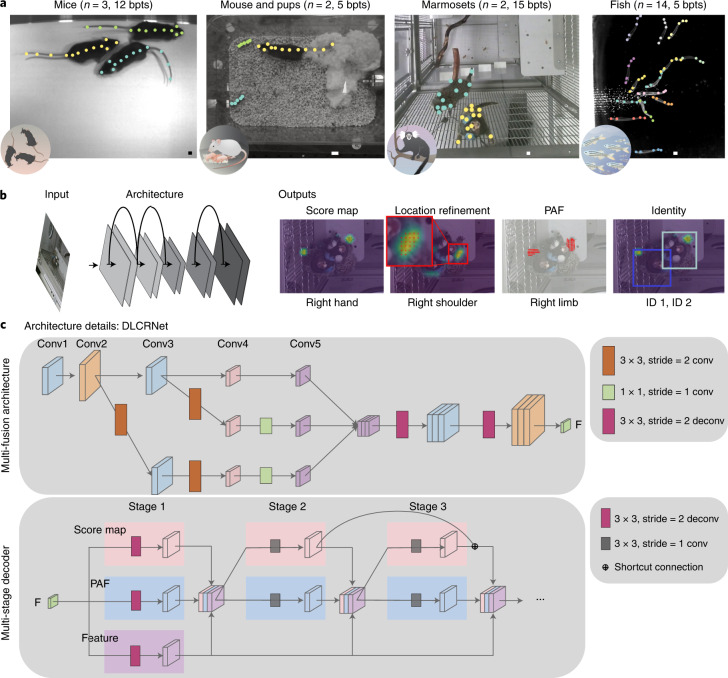
Multi-animal DeepLabCut architecture and benchmarking datasets. **a**, Example (cropped) images with (manual) annotations for the four datasets: mice in an open field arena, parenting mice, pairs of marmosets and schooling fish. bpts, body parts. Scale bars, 20 pixels. **b**, A schematic of the general pose estimation module. The architecture is trained to predict the keypoint locations, PAFs and animal identity. Three output layers per keypoint predict the probability that a joint is in a particular pixel (score map) as well as shifts in relation to the discretized output map (location refinement field). Furthermore, PAFs predict vector fields encoding the orientation of a connection between two keypoints. Example predictions are overlaid on the corresponding (cropped) marmoset frame. The PAF for the right limb helps linking the right hand and shoulder keypoints to the correct individual. **c**, Our architecture contains a multi-fusion module and a multi-stage decoder. In the multi-fusion module, we add the high-resolution representation (conv2, conv3) to low-resolution representation (conv5). The features from conv2 and conv3 are downsampled by two and one 3 × 3 convolution layer, respectively to match the resolution of conv5. Before concatenation the features are downsampled by a 1 × 1 convolution layer to reduce computational costs and (spatially) upsampled by two stacked 3 × 3 deconvolution layers with stride 2. The multi-stage decoder predicts score maps and PAFs. At the first stage, the feature map from the multi-fusion module are upsampled by a 3 × 3 deconvolution layer with stride 2, to get the score map, PAF and the upsampled feature. In the latter stages, the predictions from the two branches (score maps and PAFs), along with the upsampled feature are concatenated for the next stage. We applied a shortcut connection between the consecutive stage of the score map. The shown variant of DLCRNet has overall stride 2 (in general, this can be modulated from 2 to 8).

**Extended Data Fig. 2:**
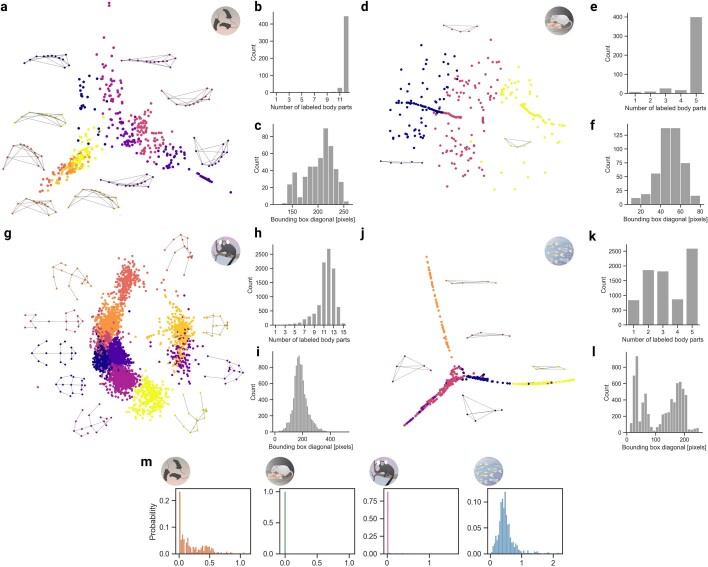
Dataset characteristics and statistics. For each datasets, normalized animal poses were clustered using K-means adapted for missing elements, and embedded non-linearly in 2D space via Isometric mapping (Tenenbaum et al. 2000). Embeedings as well as representative poses are shown for the tri-mouse dataset (a). Counts of labeled keypoints (b) and distribution of bounding box diagonal lengths (c). (d-l) show the same for the other three datsets. The Proximity Index (m) reflects the crowdedness of the various dataset scenes. Statistics were computed from the ground truth test video annotations. The mice and fish datasets are more cluttered on average than the pups and marmosets.

We considered four multi-animal experiments to broadly validate our approach: three mice in an open field, home-cage parenting in mice, pairs of marmosets housed in a large enclosure and 14 fish in a flow tank. These datasets encompass a wide range of behaviors, presenting difficult and unique computational challenges to pose estimation and tracking (Fig. 1a and Extended Data Fig. 2). The three mice frequently contact and occlude one another. The parenting dataset contained a single adult mouse with unique keypoints in close interaction with two pups hardly distinguishable from the background or the cotton nest, which also leads to occlusions. The marmoset dataset comprises periods of occlusion, close interactions, nonstationary behavior, motion blur and changes in scale. Likewise, the fish school along all dimensions of the tank, hiding each other in cluttered scenes, and occasionally leaving the camera’s field of view. We annotated 5–15 body parts of interest depending on the dataset (Fig. 1a and Extended Data Fig. 1), in multiple frames for cross-validating the pose estimation and assembly performance, as well as semiautomatically annotated several videos for evaluating the tracking performance (Table 1). For analyses, we created a random split of images plus annotations into 70% train and 30% test sets.

**Table 1:** Multi-animal pose estimation dataset characteristics

Feature	Mouse	Pups	Marmosets	Fish
Labeled frames	161	542	7,600	100
Keypoints	12	5 (+12)	15	5
Individuals	3	2 (+1)	2	14
GT identity	No	No	Yes	No
Annotated video frames	11,645	2,670	15,000	1,100
Total duration (s)	385	180	600	36

Number of labeled training frames, keypoints and individuals. Keypoint number in brackets relate to the unique animal in the frame, and unique individual in brackets is noted, that is, one parenting mouse. Animal identity was only annotated for the marmosets. For tracking, separate videos are used and the total number of densely human-annotated video frames (and their combined duration in seconds) is also indicated.

### Multi-task convolutional architectures

We developed multi-task convolutional neural networks (CNNs) that perform pose estimation by localizing keypoints in images. This is achieved by predicting score maps, which encode the probability that a keypoint occurs at a particular location, as well as location refinement fields that predict offsets to mitigate quantization errors due to downsampled score maps^13,19,20^. Then, to assemble keypoints into the grouping that defines an animal, we designed the networks to also predict ‘limbs’, that is, PAFs. This task, which is achieved via additional deconvolution layers, is inspired by OpenPose^9^. The intuition is that in scenarios where multiple animals are present in the scene, learning to predict the location and orientation of limbs will help group pairs of keypoints belonging to an individual. Moreover, we also introduce an output that allows for animal reidentification (reID) from visual input directly. This is important in the event of animals that are untrackable using temporal information alone, for example, when exiting or re-entering the scene (Fig. 1b).

Specifically, we adapted ImageNet-pretrained ResNets^22^, EfficientNets^21,23^, as well as developed a multi-scale architecture (which we call DLCRNet_ms5, Fig. 1c). We then use customized multiple parallel deconvolution layers to predict the location of keypoints as well as what keypoints are connected in a given animal (Fig. 1b). Ground truth data of annotated keypoints are used to calculate target score maps, location refinement maps, PAFs and to train the network to predict those outputs for a given input image (Fig. 1b,c) with augmentation.

### Keypoint detection and part affinity performance

**Fig. 2:**
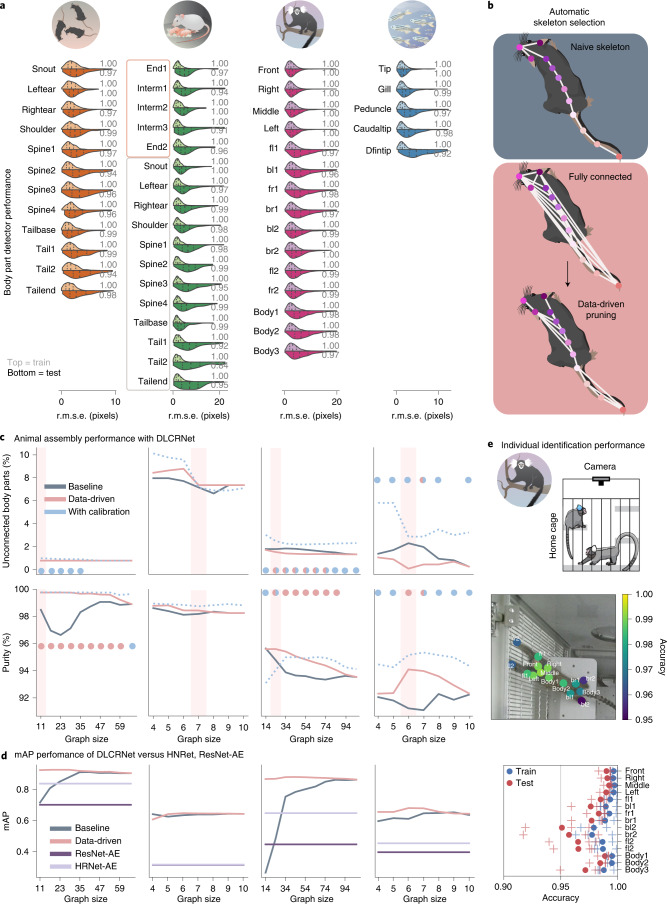
Multi-animal DeepLabCut keypoint detection and whole-body assembly performance. **a**, Distribution of keypoint prediction error for DLCRNet_ms5 with stride 8 (70% train and 30% test split). Violin plots display train (top) and test (bottom) errors. Vertical dotted lines are the first, second and third quartiles. Median test errors were 2.69, 5.62, 4.65 and 2.80 pixels for the illustrated datasets, in order. Gray numbers indicate PCK. Only the first five keypoints of the parenting dataset belong to the pups; the 12 others are keypoints of the adult mouse. **b**, Illustration of our data-driven skeleton selection algorithm. Mouse cartoon adapted with permission from ref. ^29^ under a Creative Commons licence (https://creativecommons.org/licenses/by/4.0/). **c**, Animal assembly quality as a function of part affinity graph (skeleton) size for baseline (user-defined) versus data-driven skeleton definitions. The top row displays the fraction of keypoints left unconnected after assembly, whereas the bottom row designates the accuracy of their grouping into distinct animals. The colored dots mark statistically significant interactions (two-way, repeated-measures ANOVA; see Supplementary Tables 1–4 for full statistics). Light red vertical bars highlight the graph automatically selected. **d**, mAP as a function of graph size. Shown on test data held out from 70% train and 30% test splits. The associative embedding method does not rely on a graph. The performance of MMPose’s implementation of ResNet-AE and HRNet-AE bottom-up variants is shown for comparison against our multi-stage architecture DLCRNet_ms5, here called Baseline. Data-driven is Baseline plus calibration method (one-way ANOVA show significant effects of the model: *P* values, tri-mouse 8.8 × 10^−8^, pups 6.5 × 10^−13^, marmosets 3.8 × 10^−11^, fish 4.0 × 10^−12^). **e**, Marmoset ID–Example test image together with overlaid animal identity prediction accuracy per keypoint averaged over all test images and test splits. With ResNet50_stride8, accuracy peaks at 99.2% for keypoints near the head and drops to only 95.1% for more distal parts. In the lower panel, plus signs denote individual splits, circles show the averages.

**Extended Data Fig. 3:**
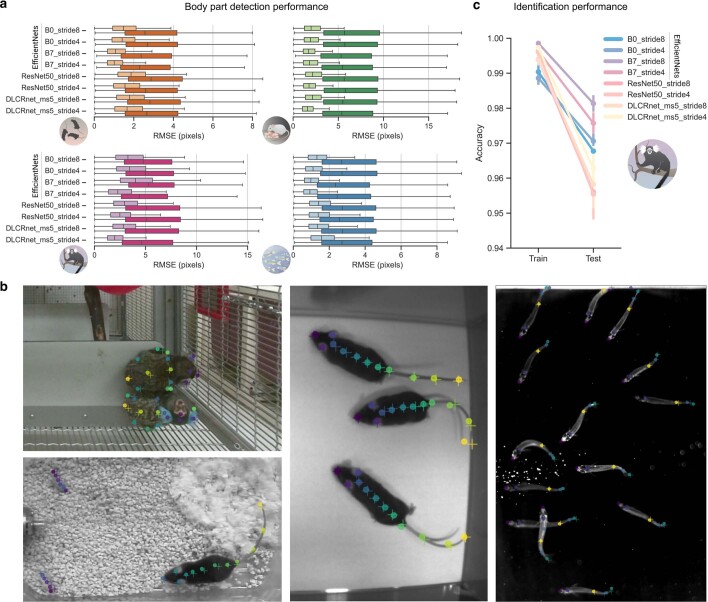
Performance of various DeepLabCut network architectures. (a) Overall keypoint prediction errors of ResNets-50 and the EfficientNets backbones (B0/B7), DLCRNet at stride 4 and 8. Distribution of train and test errors are displayed as light and dark box plots, respectively. Box plots show median, first and third quartiles, with whiskers extending past the low and high quartiles to ± 1.5 times the interquartile range. All models were trained for 60k iterations. n=independent image samples as follows: for train∣test per dataset: 112∣49 (tri-mouse); 379∣163 (pups); 5316∣2278 (marmosets); 70∣30 (fish). (b): Images on held-out test data, where plus indicates human ground truth, and the circle indicates the model prediction (shown for ResNet50 with stride 8). (c): Marmoset identification train-test accuracy for various backbones.

After an extensive architecture search (http://maDLCopt.deeplabcut.org and Extended Data Fig. 3), we demonstrate that the new DLCRNet performs very well for localizing keypoints (Fig. 2a). Specifically, we trained independent networks for each dataset, and each split, and evaluated their performance. For each frame and keypoint, we calculated the root-mean squared error (r.m.s.e.) between the detections and their closest ground truth neighbors. All the keypoint detectors performed well (DLCRNet_ms5, median test errors of 2.65, 5.25, 4.59 and 2.72 pixels for the tri-mouse, parenting, marmoset and fish datasets, respectively, Fig. 2a). The scales of these data are shown in Fig. 1a). To ease interpretation, errors were also normalized to 33% of the tip–gill distance for the fish dataset and 33% of the left-to-right ear distance for the remaining ones (Methods). We found that 93.6 ± 6.9% of the predictions on the test images were within those ranges (Fig. 2a).

**Extended Data Fig. 4:**
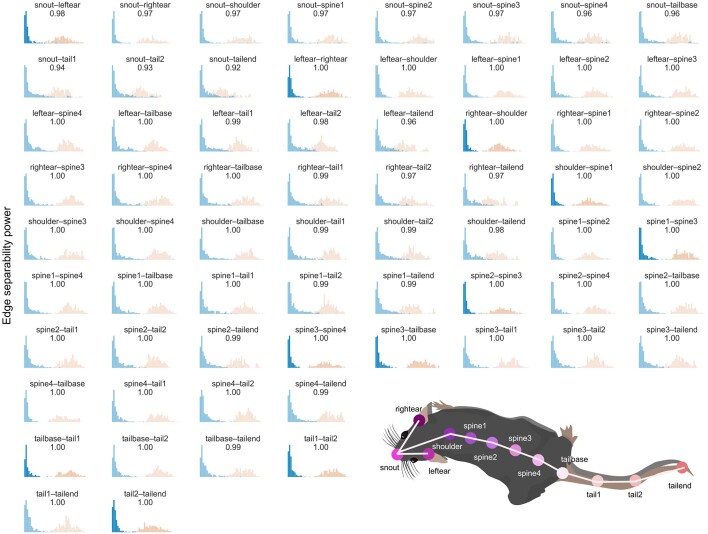
Discriminability of part affinity fields. Within- (pink) and between-animal (blue) affinity cost distributions for all edges of the mouse skeleton with DLCRNet_ms5. The saturated subplots highlight the 11 edges kept to form the smallest, optimal part affinity graph (see Fig. 2b). This is based on the separability power of an edge, that is, its ability to discriminate a connection between two keypoints effectively belonging to the same animal from the wrong ones, and reflected by the corresponding AUC scores (listed at the top of the subplots).

After detection, keypoints need to be assigned to individuals. We evaluated whether the learned PAFs helped decide whether two body parts belong to the same or different animals. For example, 66 different edges can be formed from the 12 mouse body parts and many provide high discriminability (Extended Data Fig. 4). We indeed found that predicted limbs were powerful at distinguishing a pair of keypoints belonging to an animal from other (incorrect) pairs linking different mice, as measured by a high auROC (area under the receiver operating characteristic) score (mean ± s.d. 0.99 ± 0.02).

### Data-driven individual assembly performance

Any limb-based assembly approach requires a ‘skeleton’, that is, a list of keypoint connections that allows the algorithm to computationally infer which body parts belong together. Naturally, there has to be a path within this skeleton connecting any two body parts, otherwise the body parts cannot be grouped into one animal. Given the combinatorial nature of skeletons, how should they be designed? We circumvented the need for arbitrary, hand-crafted skeletons by developing a method that is agnostic to an animal’s morphology and does not require any user input.

**Extended Data Fig. 5:**
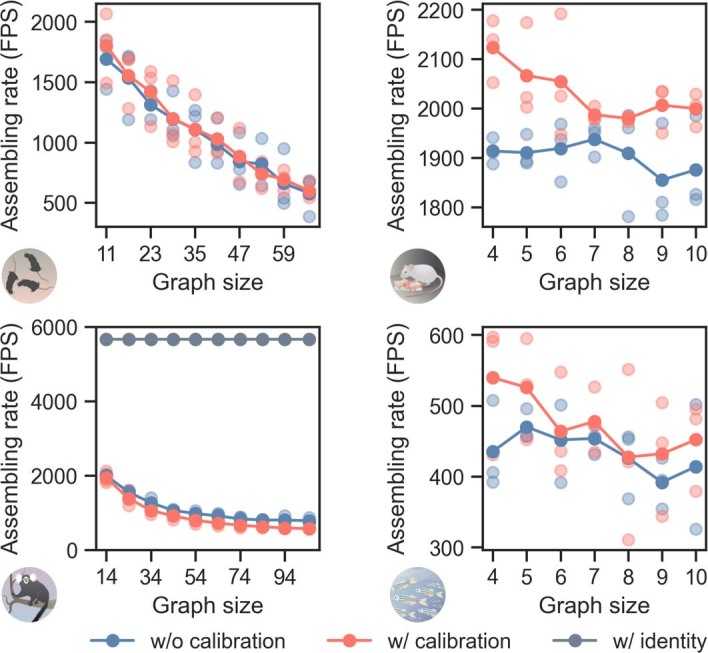
Average animal assembly speed in frames per second as a function of graph size. Assembly rates vs. graphs size for the four datasets. Improving the assembly robustness via calibration with labeled data in large graphs incurs no extra computational cost at best, and a slowdown by 25% at worst; remarkably, it is found to accelerate assembly speed in small graphs. Relying exclusively on keypoint identity prediction results in average speeds of around 5600 frames per second, independent of graph size. Three timing experiments were run per graph size (lighter colored dots) and averages are shown. Note that assembling rates exclude CNN processing times. Speed benchmark was run on a workstation with an Intel(R) Core(TM) i9-10900X CPU 3.70GHz.

We devised a data-driven method where the network is first trained to predict all graph edges and the least discriminative edges (for deciding body part ownership) are not used at test time to determine the optimal skeleton. We found that this approach yields skeletons with fewer errors (unconnected body parts and with higher purity, Fig. 2b,c) and it improves performance. Crucially, it means users do not need to design any skeletons. Our data-driven method (with DLCRNet_ms5) outperforms the naive (baseline) method, enhances ‘purity of the assembly’: that is, the fraction of keypoints that were grouped correctly per individual (Supplementary Table 1), and reduces the number of missing keypoints (Supplementary Table 2). Comparisons revealed significantly higher assembly purity with automatic skeleton pruning versus a naive skeleton definition at most graph sizes, with respective gains of up to 3.0, 2.0 and 2.4 percentage points in the tri-mouse (two-way repeated measure analyses of variance (ANOVA): graph size 23; *P* < 0.001), marmosets (graph size 34, *P* = 0.002) and fish datasets (graph size 6, *P* < 0.001) (Fig. 2b,c). Furthermore, to accommodate diverse body plans and annotated keypoints for different animals and experiments, our inference algorithm works for arbitrary graphs. Animal assembly achieves at least 400 frames per second in scenes with 14 animals, and up to 2,000 for small skeletons in two or three animals (Extended Data Fig. 5).

**Extended Data Fig. 6:**
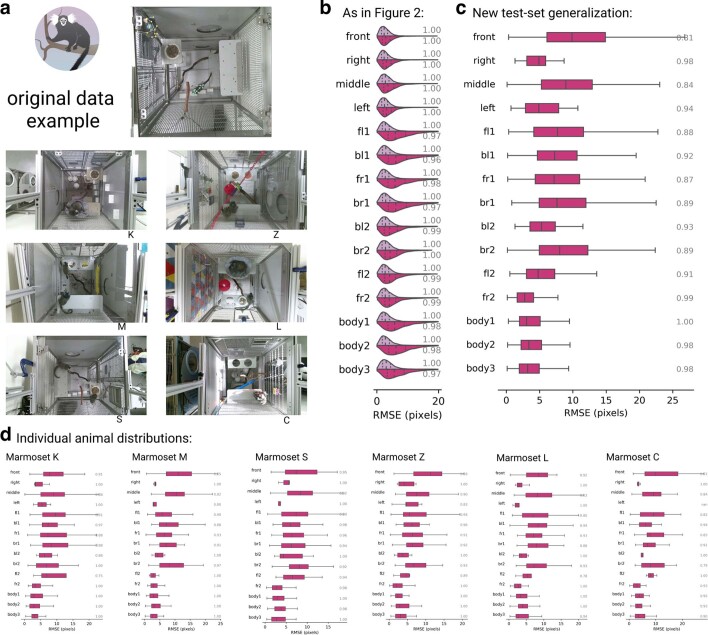
Performance on out of domain marmoset data. (a) Example images from original dataset, and example generalization test images. (b) Median RMSE and PCK (gray numbers) for data and network (DLCRNet) as shown in Fig. 2a. (c) same, but on the generalization test images (n=300) (d) same but per cage as shown in a (n=30 test images per marmoset). Box plots show median, first and third quartiles, with whiskers extending past the low and high quartiles to ± 1.5 times the interquartile range.

To additionally benchmark our network and assembly contributions, we compared them to methods that achieve state-of-the art performance on COCO^18^, a challenging, large-scale multi-human pose estimation benchmark. Specifically, we considered HRNet-AE and ResNet-AE. Our models performed significantly better than these state-of-the-art methods (one-way ANOVA: *P* values, tri-mouse 8.8 × 10^−08^, pups 6.5 × 10^−13^, marmosets 3.8 × 10^−11^, fish 4.0 × 10^−12^, Fig. 2d) on all four animal benchmark datasets. Last, while the datasets themselves contain diverse animal behaviors, and only 70% is used to train, as an additional test of generalization we used ten held-out marmoset videos that came from different cages (Extended Data Fig. 6). We find in this challenging test there is a roughly 0.25 drop in mean average precision (mAP). It is known that simply adding (a fraction of the new) data into the training set alleviates such drops (reviewed in ref. ^7^).

**Extended Data Fig. 7:**
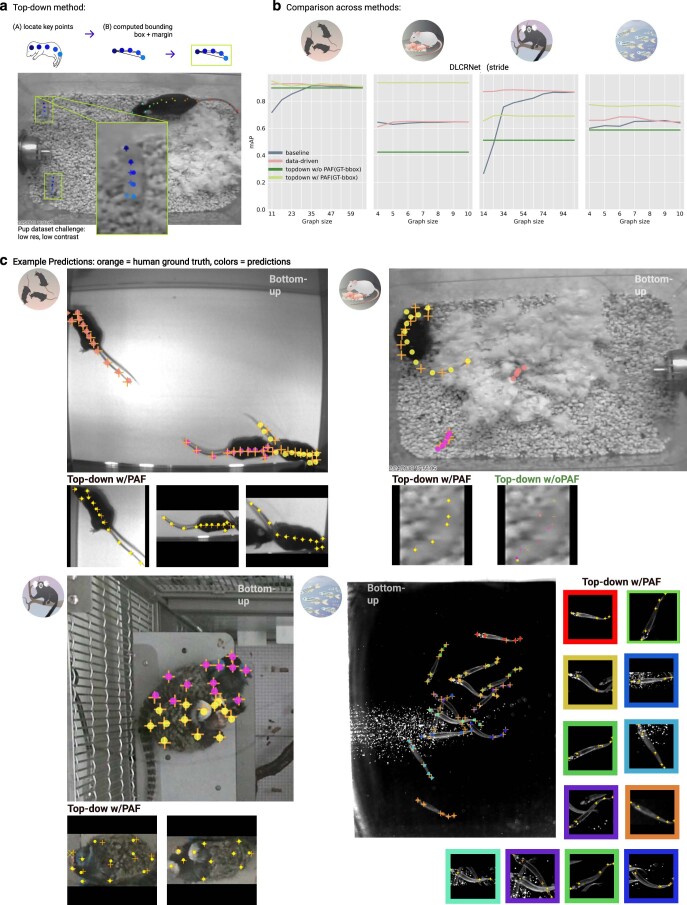
Comparison of top-down methods with and without assembly. (a) Schematics of top-down method with example images from the pup dataset, which consists of first detecting individuals and then performing pose prediction on each bounding box (plus is human ground truth, and the circle is bottom-up model (DLCRNet, stride 8, data-driven) predictions. (b) Performance mAP computed for top-down method with and without PAFs and bottom-up method (baseline, data-driven) as also shown in Fig. 2d. PAF vs. w/o PAF one-way ANOVA p-values, tri-mouse: 4.656e-11, pups: 3.62e-12, marmosets: 1.33e-28, fish: 1.645e-06). There were significant model effects across all datasets: one-way ANOVA p-values– tri-mouse: 4.13e-11, pups: 4.59e-25, marmosets: 3.04e-40, fish: 1.18e-14. (c) Example predictions within the smaller images (that is, bounded crops) from the top-down model (that is, w/PAF), and bottom-up predictions (full images, as noted).

We reasoned the strong multi-animal performance is due to the assembly algorithm based on PAFs. Therefore, we tested the performance of the network in a top-down setting with and without PAFs, that is, by considering images that are cropped around each animal (bounding boxes, Extended Data Fig. 7a). We found that our assembly algorithm significantly improves mAP performance (PAF versus without PAF one-way ANOVA *P*, tri-mouse 4.656 × 10^−11^, pups 3.62 × 10^−12^, marmosets 1.33 × 10^−28^, fish 1.645 × 10^−6^, Extended Data Fig. 7b,c). Collectively, the direct assembly to tracking (that is, the bottom-up method) is likely the optimal approach for most users as it reasons over the whole image.

### Predicting animal identity from images

Animals sometimes differ visually, for example due to distinct coat patterns, because they are marked, or carry different instruments (such as an integrated microscope^24^). To allow our method to take advantage of such scenarios and improve tracking later on, we developed a network head that learns the identity (ID) of animals with the same CNN backbone. To benchmark the ID output, we focused on the marmoset data, where (for each pair) one marmoset had light blue dye applied to its tufts. ID prediction accuracy on the test images ranged from >0.99 for the keypoints closest to the marmoset’s head to 0.95 for more distal keypoints (Fig. 2e and Extended Data Fig. 3c). Thus, DeepLabCut can reID the animal on a per-body-part basis (Fig. 2e).

### Tracking of individuals

**Fig. 3:**
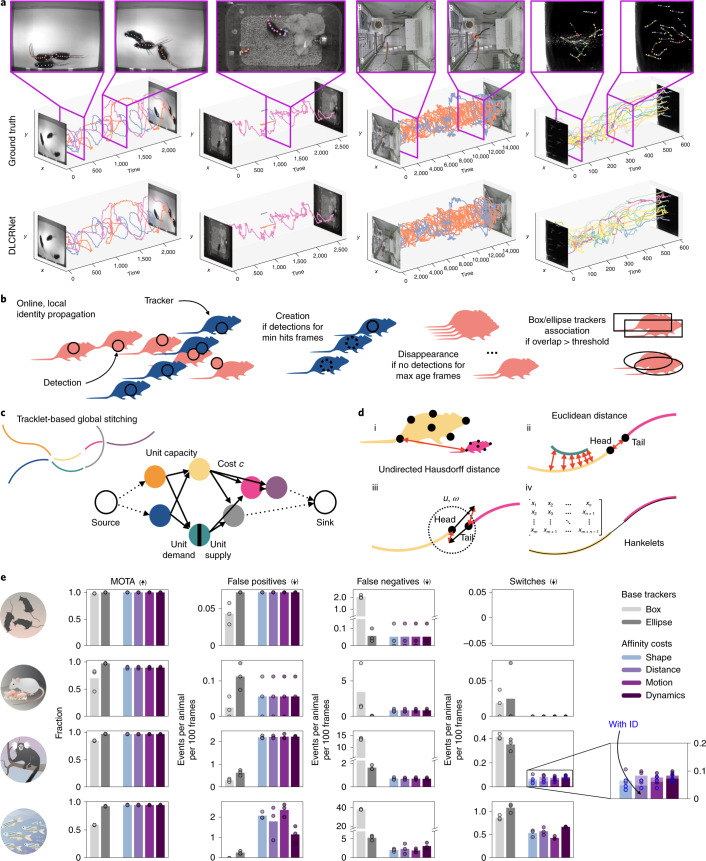
Linking whole-body assemblies across time. **a**, Ground truth and reconstructed animal tracks (with DLCRNet and ellipse tracking), together with video frames illustrating representative scene challenges. **b**, The identities of animals detected in a frame are propagated across frames using local matching between detections and trackers (with costs, ‘motion’ for all datsets and ‘distance’ for fish). **c**, Tracklets are represented as nodes of a graph, whose edges encode the likelihood that the connected pair of tracklet belongs to the same track. **d**, Four cost functions modeling the affinity between tracklets are implemented: shape similarity using the undirected Hausdorff distance between finite sets of keypoints (i); spatial proximity in Euclidean space (ii); motion affinity using bidirectional prediction of a tracklet’s location (iii); and dynamic similarity via Hankelets and time-delay embedding of a tracklet’s centroid (iv). **e**, Tracklet stitching performance versus box and ellipse tracker baselines (arrows indicate if higher or lower number is better), using MOTA, as well as rates of false negative (FN), false positives (FP) and identity switch expressed in events per animal and per sequence of 100 frames. Inset shows that incorporating appearance/identity prediction in the stitching further reduces the number of switches and improves full track reconstruction. Total number of frames: tri-mouse, 2,330; parenting, 2,670; marmosets, 15,000 and fish, 601.

Once keypoints are assembled into individual animals, the next step is to link them temporally. To measure performance in the next steps, entire videos (one from each dataset) are manually refined to form ground truth sequences (Fig. 3a and Table 1). Reasoning over the whole video for tracking individuals is not only computationally costly, but also unnecessary. For instance, when animals are far apart, it is straightforward to link each one correctly across time. Thus, we devised a divide-and-conquer strategy. We use a simple, online tracking approach to form reliable ‘tracklets’ from detected animals in adjacent frames. Difficult cases (for example, when animals are closely interacting or after occlusion) often interrupt the tracklets, causing ambiguous fragments that cannot be easily temporally linked. We address this crucial issue post hoc by optimally stitching tracklets using multiple spatio-temporal cues.

Assembled animals are linked across frames to form tracklets, that is, fragments of continuous trajectories. This task entails the propagation of an animal’s identity in time by finding the optimal association between an animal and its predicted location in the adjacent frame (Fig. 3b–d). The prediction is made by a lightweight ‘tracker’. In particular, we implemented a box and an ellipse tracker. Whereas the former is standard in the object tracking literature (for example, refs. ^25,26^), we recognized the sensitivity of its formulation to outlier detections (as it is mostly used for pedestrian tracking). Thus, the ellipse tracker was developed to provide a finer parametrization of an animal’s geometry. Overall, the ellipse tracker behaves better than the box tracker, reaching near-perfect multi-object tracking accuracy (MOTA) (0.78 versus 0.97) and producing on average 92% less false negatives; no differences in the switch rate was observed (Fig. 3e).

Because of occlusions, dissimilarity between an animal and its predicted state, or other challenging yet common multi-animal tracking issues, tracklets can be interrupted and therefore rarely form complete tracks across a video. The remaining challenge therefore is to stitch these sparse tracklets so as to guarantee continuity and kinematic consistency. Our approach is to cast this task as a global minimization problem, where connecting two candidate tracklets incurs a cost inversely proportional to the likelihood that they belong to the same track. Advantageously, the problem can now be elegantly solved using optimization techniques on graph and affinity models (Fig. 3c,d).

Compared to only local tracking, we find that our stitching method reduces switches, even in the challenging fish and marmosets datasets (average reduction compared to local ellipse tracking, 63%; Fig. 3e). To handle a wide range of scenarios, multiple cost functions are devised to model the affinity between a pair of tracklets based on their shape, proximity, motion, dynamics and/or appearance (below and Supplementary Videos 1–4). Last, to allow users to understand the error rate and correct errors, we developed a Refine Tracklets GUI. Here, we leverage confidence of the tracking to flag sequences of frames that might need attention, namely when swaps might occur (Extended Data Fig. 1b).

**Extended Data Fig. 8:**
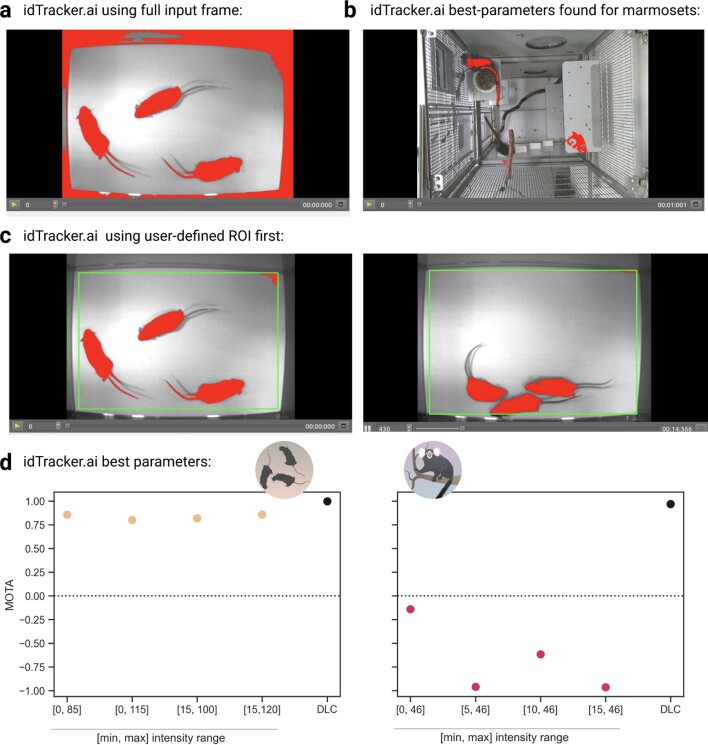
Performance of idtracker.ai. (a) Segmented regions (red) overlaid on example image in idtracker.ai GUI. Example how idtracker.ai fails to segment only the mice in the full data frame for tri-mouse and (b) in marmoset dataset. (c) Using the ROI selection feature, we could find mostly just mice. However, due to the inhomogeneous lighting, the segmentation is not error-free. (d) Result of a grid search to find optimal parameters for idtracker with MOTA scores on the same videos as shown in Fig. 3a,e; one-sided, one-sample T-tests indicated that idtracker.ai performed significantly worse than DeepLabCut in both datasets (tri-mouse: T=-11.03, p=0.0008, d=5.52; marmosets: T=-8.43, p=0.0018, d=4.22).

Other recent methods for tracking animals have been proposed, such as idtracker.ai^27^. While this tool does not perform pose estimation, we wanted to specifically compare tracking performance. We attempted to use the easiest (tri-mouse) and marked-animal (marmoset) datasets with idtracker.ai. After an extensive grid search for hyperparameters, only the tri-mouse mice dataset could be reliably tracked, yet the performance of our method was significantly better (one-sided, one-sample *t*-tests indicated that idtracker performed significantly worse than DeepLabCut in both datasets (tri-mouse *t* = −11.03, *P* = 0.0008, *d* = 5.52; marmosets *t* = −8.43, *P* = 0.0018, *d* = 4.22: Supplementary Video 5 and Extended Data Fig. 8).

**Extended Data Fig. 9:**
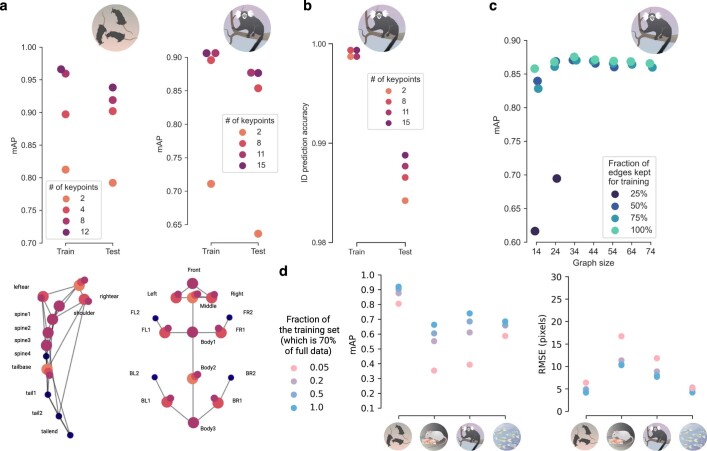
Parameter sensitivity: Evaluation of number of body parts, frames, and PAF sizes. (a) The number of keypoints affects mAP; evaluated with ResNet50 stride8 on the two datasets with the most keypoints originally labeled by subsampling the keypoints. [Mouse: snout/tailbase (2) + leftear/rightear (4) + shoulder/spine1/spine2/spine3 (8) vs. full (12); Marmoset: Middle/Body2 (2) + FL1/BL1 /FR1/BR1/Left/Right (8) + front/body1/body3 (11) vs. full (15)] (b) Identity prediction is not strongly affected by the number of keypoints used (same experiments as in a, but for identity). (c) Impact of graph size, and randomly dropping edges on performance. (d) Test performance on 30% of the data vs. training set size (as fraction of 70%) for all four datasets.

Note, for keypoint selection we remain fully agnostic to the user-defined inputs, giving the user freedom over what keypoints ultimately serve their research, but we do guide the user by showing them how such decisions could affect performance (Extended Data Fig. 9).

### Leveraging animal ID and reID in tracking

**Fig. 4:**
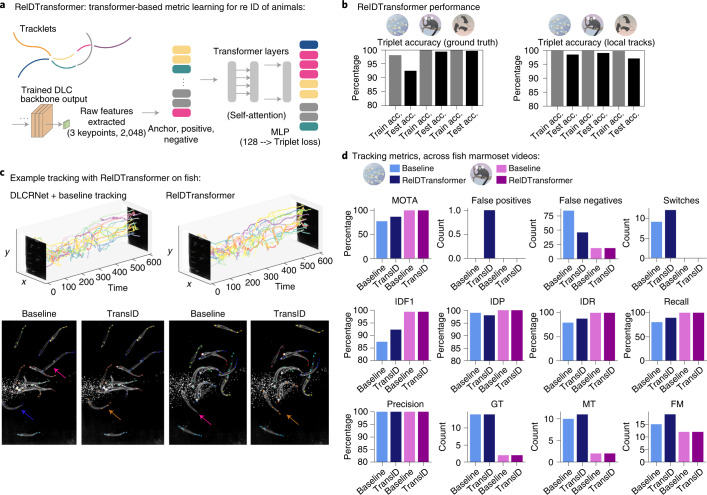
Unsupervised reID of animals. **a**, Schematic of the transformer architecture we adapted to take pose-tensor outputs of the DeepLabCut backbone. We trained it with triplets sampled from tracklets and tracks. **b**, Performance of the ReIDTransformer method on unmarked fish, mice and marked marmosets. Triplet accuracy (acc.) is reported for triplets sampled from ground truth (GT) tracks and local tracklets only. We used only the top 5% of the most crowded frames, as those are the most challenging. **c**, Example performance on the challenging fish data. Top: fish-identity-colored tracks. Time is given in frame number. Bottom: example frames (early versus later) from baseline or ReIDTransformer. Arrows highlight performance with ReIDTransformer: pink arrows show misses; orange shows correct ID across frames in ReIDTransformer versus blue to orange in baseline. **d**, Tracking metrics on the most crowded 5% of frames (30 frames for fish, 744 for marmosets, giving 420 fish targets and 1,488 marmoset targets); computed as described in Methods. IDF1, ID measure, global min-cost F1 score; IDP, ID measure, global min-cost precision; IDR, ID measures: global min-cost recall; Recall, number of detections over number of objects; Precision, number of detected objects over sum of detected and false positives; GT, number of unique objects; MT, mostly tracked and FM, number of fragmentations.

When animals can disappear from the field of view, they cannot be tracked by temporal association alone and appearance cues are necessary. Indeed, for the marmosets, incorporating visual appearance learned in a supervised fashion, further reduced the number of switches by 26% (Fig. 3e). Additionally, we next considered the case with animals that are not clearly distinguishable to the human annotator, thus no ground truth can be easily provided. To tackle this challenge, we introduce an unsupervised method way based on transformers to learn animal ID via metric learning (Fig. 4a–c and Methods). This provides up to a 10% boost in MOTA performance in the very challenging fish data, particularly in difficult sequences (Fig. 4d).

### Social marmosets

**Fig. 5:**
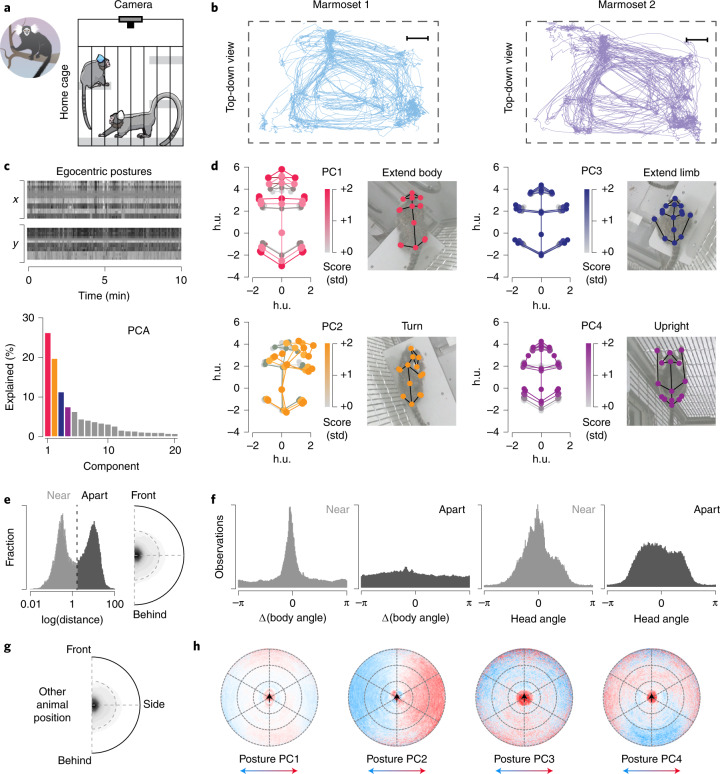
Application to multi-marmoset social behaviors. **a**, Schematic of the marmoset recording setup. **b**, Example tracks, 30 min plotted from each marmoset. Scale bars, 0.2 m. **c**, Example egocentric posture data, where the ‘Body2’ point is (0,0) and the angle formed by ‘Body1’ and ‘Body3’ is rotated to 0°. We performed principal component analysis on the pooled data of both marmosets for all data. **d**, Average postures along each principal component; note that only one side of the distribution is represented in the image (that is, 0 to 2 instead of −2 to 2). **e**, Histogram of log-distance between a pair of marmosets normalized to ear-center distance. **f**, Computed body angle versus observation count. **g**, Density plot of where another marmoset is located relative to marmoset 1. **h**, Postural principal components (from **d**) as a function of the relative location of the other marmoset. Thereby, each point represents the average postural component score for marmoset 1 when marmoset 2 is at that point. h.u., head units.

Finally, we demonstrate a use-case of multi-animal DeepLabCut by analyzing 9 h (824,568 frames) of home-cage behavior of pairs of marmosets (Fig. 5a,b). We tracked by ReID on a frame-by-frame basis versus only using tracklet information. We found that the marmosets display diverse postures that are captured by principal component analysis on egocentrically aligned poses (Fig. 5c,d). Furthermore, we found that when the animals are close, their bodies tend to be aligned and they tend to look in similar directions (Fig. 5e,f). Finally, we related the posture and the spatial relationship between the animals and found a nonrandom distribution. For instance, marmosets tended to face the other animal when apart (Fig. 5g,h). Thus, DeepLabCut can be used to study complex social interactions over long timescales.

## Discussion

Here we introduced a multi-animal pose estimation and tracking system thereby extending DeepLabCut^19–21^. We developed and leveraged more powerful CNNs (DLCRNet) that we show have strong performance for animal pose estimation. Due to the variable body shapes of animals, we developed a data-driven way to automatically find the best skeleton for animal assembly, and we designed fast trackers that also reason over long timescales and are more robust to the body plan, and outperform tailored animal tracking methods such as idTracker.ai. Our method is flexible and not only deals with multiple animals (with the same body plan), but also with one agent dealing with multiple others (as with the parenting mouse, where there are identical looking pups, but a unique adult mouse).

For animal pose and tracking, leveraging identity can be an important tool^4^. We introduce ways to allow both marked animals (supervised) and unsupervised animal identity tracking. For marked animals this means if users consistently label a known feature across the dataset (such as the blue tufts of the 40 pairs of marmosets included in labeled data, or consistent marker on a mouse’s tail), they can simply input the identity ‘true’ (or check the box in the GUI) in DeepLabCut and this trains an ID head. If they are not marked, users could use the reID tracking method that performs unsupervised identity estimation. Thereby, our framework integrates various costs related to movement statistics and the learned animal identity.

Open-access benchmark datasets are critical to collectively advance tools^7^. Here, we open-source four datasets that pose different challenges. As we show, there is little room for improvement on the tri-mouse data, but the schooling fish is not a solved problem. Thus, while our method is fast, generally robust, and can leverage identity of animals to enhance pose-tracking performance, we believe the benchmark can also spur progress on how to improve performance for occluded, similar looking animals.

We developed both bottom-up and top-down variants for multi-animal DeepLabCut (maDLC), allowing the user an extended selection of methods (Supplementary Note 1). While our results suggest that the bottom-up pipeline should be the default, depending on the application, top-down approaches might be better suited. Both classes have limitations and features (reviewed in ref. ^7^).

In this work, we strive to develop high-performance code that requires limited user input yet flexibility. In the code, we provide 3D support for multi-animal pose estimation (via multi-camera use), plus this multi-animal variant can be integrated with our real-time software, DeepLabCut-Live!^28^. Another important user input is at the stage of tracking, where users can input how many animals should be identified in a given video. In this paper, we test up to 14 animals, but this is not a hard upper limit. One ‘upper limit’ is ultimately the camera resolution, as one needs to be able to localize keypoints of animals. Thus, if animals are very small, other tracking tools might be better suited. From pose, to optimal skeleton selection, to tracking: all of the outlined steps can be run in ten lines of code or all from a GUI such that zero programming is required (https://deeplabcut.github.io/DeepLabCut).

## Methods

### Tri-mouse dataset

Three wild-type (C57BL/6J) male mice ran on a paper spool following odor trails^19^. These experiments were carried out in the laboratory of V.N. Murthy at Harvard University (temperature of housing was 20–25 °C, humidity 20–50%). Data were recorded at 30 Hz with 640 × 480 pixels resolution acquired with a Point Grey Firefly FMVU-03MTM-CS. One human annotator was instructed to localize the 12 keypoints (snout, left ear, right ear, shoulder, four spine points, tail base and three tail points) across 161 frames sampled from within DeepLabCut using the *k*-means clustering approach (across eight videos). All surgical and experimental procedures for mice were in accordance with the NIH Guide for the Care and Use of Laboratory Animals and approved by the Harvard Institutional Animal Care and Use Committee (IACUC).

### Parenting behavior

Parenting behavior is a pup-directed behavior observed in adult mice involving complex motor actions directed toward the benefit of the offspring^30,31^. These experiments were carried out in the laboratory of C. Dulac at Harvard University (temperature of housing was 20–25 °C, humidity 20–50%).

The behavioral assay was performed in the home-cage of single-housed adult (age was less than 180 d old) female C57Bl6/J *Mus musculus* in dark (red light only) conditions. For these videos, the adult mouse was monitored for several minutes in the cage followed by the introduction of pup (4 d old, sex unknown) in one corner of the cage. The behavior of the adult and pup was monitored for a duration of 15min. A video was recorded at 30 Hz using a Microsoft LifeCam camera (part no. 6CH-00001) with a resolution of 1,280 × 720 pixels or a Geovision camera (model no. GV-BX4700-3V) also acquired at 30 frames per second (fps) at a resolution of 704 × 480 pixels. A human annotator labeled on the adult animal the same 12 body points as in the tri-mouse dataset and five body points on the pup along its spine. Initially only the two ends were labeled, and intermediate points were added by interpolation and their positions was manually adjusted if necessary. Frames were generated from across 25 videos.

### Marmoset home-cage behavior

Videos of common marmosets (*Callithrix jacchus*) were made in the laboratory of G. Feng at MIT. Male and female marmoset pairs housed here (*n* = 50, 30 pairs, age range of 2 to 12 years) were recorded using Kinect V2 cameras (Microsoft) with a resolution of 1,080 pixels and frame rate of 30 Hz. After acquisition, images to be used for training the network were manually cropped to 1,000 × 1,000 pixels or smaller. For our analysis, we used 7,600 labeled frames from 40 different marmosets collected from three different colonies (in different facilities, thus over 20 videos were used for dataset generation). Each cage contains a pair of marmosets, where one marmoset had light blue dye applied to its tufts. One human annotator labeled the 15 body points on each animal present in the frame (frames contained either one or two animals). All animal procedures were overseen by veterinary staff of the MIT and Broad Institute Department of Comparative Medicine, in compliance with the National Institutes of Health guide for the care and use of laboratory animals and approved by the MIT and Broad Institute animal care and use committees. As a test of out-of-domain generalization, we additionally labeled 300 frames from ten new cages and animals. See Fig. 5 for example images and results.

We also analyzed two long-term recording sessions from a pairs of marmosets with the DLCRNet_ms5 model, by reidentifying each marmoset in each frame with the ID head. Overall we considered about 9 h (824,568 frames) from two different home cages. We computed the principal components for egocentric postures as well as illustrated their relative head and body orientations (Fig. 5). For Fig. 5e, the distances are normalized based on the running average distance between each ear tuft to center of head, over a second. This measurement does correlate well with depth values in videos recorded with a depth channel (which was not done for the example sessions). To make the postural data egocentric, we first centered the data around ‘Body2’ (*x* = 0, *y* = 0) and then rotated it such that the line formed by ‘Body1’, ‘Body2’ and ‘Body3’ was as close to the line ‘*x* = 0’ as possible.

### Fish schooling behavior

Schools of inland silversides (*Menidia beryllina*, *n* = 14 individuals per school, sex unknown but likely to be equal females and males, aged approximately 9 months) were recorded in the Lauder Laboratory at Harvard University while swimming at 15 speeds (0.5 to 8 body lengths per s at 0.5 body lengths per s intervals) in a flow tank with a total working section of 28 × 28 × 40 cm as described in previous work^32^, at a constant temperature (18 ± 1^∘^C) and salinity (33 parts per thousand), at a Reynolds number of approximately 10,000 (based on body length). Dorsal views of steady swimming across these speeds were recorded by high-speed video cameras (FASTCAM Mini AX50, Photron) at 60–125 fps (feeding videos at 60 fps, swimming alone 125 fps). The dorsal view was recorded above the swim tunnel and a floating Plexiglas panel at the water surface prevented surface ripples from interfering with dorsal view videos. Five keypoints were labeled (tip, gill, peduncle, dorsal fin tip, caudal tip) and taken from five videos.

### Dataset properties

All frames were labeled with the annotation GUI; depending on the dataset, between 100 and 7,600 frames were labeled (Table 1). We illustrated the diversity of the postures by clustering (Extended Data Fig. 2). To assess the level of interactions, we evaluate a Proximity Index (Extended Data Fig. 2m), whose idea is inspired by ref. ^33^ but its computation was adapted to keypoints. For each individual, instead of delineating bounding boxes to determine the vicinity of an animal we rather define a disk centered on the individual’s centroid and of sufficiently large radius such that all of that individual’s keypoints are inscribed within the disk; this is a less static description of the immediate space an animal can reach. The index is then taken as the ratio between the number of keypoints within that region that belong to other individuals and the number of keypoints of the individual of interest (Extended Data Fig. 2m).

For each dataset we created one random split with 70% of the data used for training and the rest for testing (unless otherwise noted). Note that identity prediction accuracy (Fig. 2d) and tracking performance (Fig. 3e) are reported on all three splits, and all show little variability. The data are available as a benchmark challenge at https://benchmark.deeplabcut.org/.

### Multi-task deep-learning architecture

DeepLabCut consists of keypoint detectors, comprising a deep CNN pretrained on ImageNet as a backbone together with multiple deconvolutional layers^13,19,21^. Here, as backbones we considered Residual Networks (ResNet)^22^ and EfficientNets^21,23^. Other backbones are integrated in the toolbox^21,28^ such as MobileNetV2 (ref. ^34^). We use a stride of 16 for the ResNets (achieved by atrous convolution) and then upsample the filter banks by a factor of two to predict the score maps and location refinement fields with an overall stride of eight. Furthermore, we developed a multi-scale architecture that upsamples from conv5 and fuses those filters with filters learned as 1 × 1 convolutions from conv3. This bank is then upsampled by a factor of two via deconvolution layers. This architecture thus learns from multiple scales with an overall stride of four (including the upsampling in the decoder). We implemented a similar architecture for EfficientNets. These architectures are called ResNet50_strideX and (EfficientNet) bY_strideX for strides four to eight; we used ResNet50 as well as EfficientNets B0 and B7 for experiments (Extended Data Fig. 3).

We further developed a multi-scale architecture (DLCRNet_ms5) that fuses high-resolution feature map to lower resolution feature map (Fig. 1c)—we concatenated the feature map from conv5, the feature map learned as a 3 × 3 convolutions followed by a 1 × 1 convolutions from conv3 and the feature map learned as 2 stacked 3 × 3 convolutions and a 1 × 1 convolutions from conv2. This bank is then upsampled via (up to) two deconvolution layers. Depending on how many deconvolution layers are used this architecture learns from multiple scales with an overall stride of 2–8 (including the upsampling in the decoder). Note, during our development phase we used 95% train and 5% test splits of the data; this testing is reported at http://maDLCopt.deeplabcut.org and in our preprint^35^.

DeepLabCut creates three output layers per keypoint that encode an intensity and a vector field. The purpose of the deconvolution layers is to upsample the spatial information (Fig. 1b,c). Consider an input image *I*(*x*,*y*) with ground truth keypoint (*x*^*k*^,*y*^*k*^) for index *k*. One of the output layers encodes the confidence of a keypoint *k* being in a particular location (*S*^*k*^(*p*,*q*)), and the other two layers encode the (*x*-) and (*y*-) difference (in pixels of the full-sized) image between the original location and the corresponding location in the downsampled (by the overall stride) location (${L}_{x}^{k}(p,q)$ and ${L}_{y}^{k}(p,q)$). For each training image the architecture is trained end-to-end to predict those outputs. Thereby, the ground truth keypoint is mapped into a target score map, which is 1 for pixels closer to the target (this can be subpixel location) than radius *r* and 0 otherwise. We minimize the cross-entropy loss for the score map (*S*^*k*^) and the location refinement loss was calculated as a Huber loss^13,19^.

To link specific keypoints within one animal, we use PAFs, which were proposed by Cao et al.^9^. Each (ground truth) PAF ${P}_{x}^{l}(p,q)$ and ${P}_{y}^{l}(p,q)$ for limb *l* connecting keypoint *k*_*i*_ and *k*_*j*_ places a directional unit vector at every pixel vector within a predefined distance from the ideal line connecting two keypoints (modulated by pafwidth). We trained DeepLabCut to also minimize the *L*1-loss between the predicted and true PAFs, which is added to the other losses.

Inspired by Cao et al.^9^, we refine the score maps and PAFs in multiple stages. As can be seen from Fig. 1b, at the first stage, the original image feature from the backbone is fed into the network to predict the score map, PAF and the feature map. The output of each branch, concatenated with the feature map is fed into the subsequent stages. However, unlike Cao et al., we observed that simply adding more stages can cause performance degradation. To overcome that, we introduced shortcut connections between two consequence stages on the score map branch to improve multiple stage prediction.

Examples for score maps, location refinement and PAFs are shown in Fig. 1b. For training, we used the Adam optimizer^36^ with learning schedule (0.0001 for first 7,500 iterations then 5 × 10^−5^ until 12,000 iterations and then 1 × 10^−5^) unless otherwise noted. We trained for 60,000–200,000 (for the marmosets) iterations with batch size 8; this was enough to reach good performance (Fig. 2a and Extended Data Fig. 3). During training we also augmented images by using techniques including rotation, covering with random boxes and motion blur. We also developed a keypoint-aware image cropping technique to occasionally augment regions of the image that are dense in keypoints. Crop centers are sampled applying at random one of the following two strategies: uniform sampling over the whole image; or sampling based on keypoint density, where the probability of a point being sampled increases in proportion to its number of neighbors (within a radius equal to 10% of the smallest image side). Crop centers are further shifted along both dimensions by random amounts no greater than 40% of the crop size—the hyperparameters can be changed by the user.

### CNN-based identity prediction

For animal identification we used a classification approach^4^, while also considering spatial information. To have a monolithic solution (with just a single CNN), we simply predict in parallel the identity of each animal from the image. For this purpose, *n* deconvolution layers are added for *n* individuals. The network is trained to predict the summed score map for all keypoints of that individual. At test time, we then look up which of the output classes has the highest likelihood (for a given keypoint) and assign that identity to the keypoint. This output is trained jointly in a multi-task configuration. We evaluate the performance for identity prediction on the marmoset dataset (Fig. 2e).

Identity prediction can be leveraged by DeepLabCut in three different ways: (1) for assembly, by grouping keypoints based on their predicted identity; (2) for local, frame-by-frame tracking, using a soft-voting scheme where body parts are regarded as individual classifiers providing an identity probability and (3) for global stitching, by weighing down the cost of edges connecting two tracklets of similar appearance (as in Figs. 3 and 4). These three sequential stages can thus be made reliant on visual appearance features alone, as done with the long recordings of marmoset behavior (Fig. 5).

### Multi-animal inference

Any number of keypoints can be defined and labeled with the toolbox; additional ones can be added later on. Based on our experience and testing, we recommend labeling more keypoints than a subsequent analysis might require, since it improves the part detectors^19^ and, more importantly, animal assembly (Extended Data Fig. 9a).

Before decoding, score maps are smoothed with a Gaussian kernel of spread *σ* = 1 to make peaks more salient^37^. For each keypoint one obtains the most likely keypoint location (x^*^,y^*^) by taking the maximum: (p^*^,q^*^) = argmax_(*p*,*q*)_*S*^*k*^(*p*,*q*) and computing:

1 $$\begin{array}{l}{x}^{* }={p}^{* }\cdot \lambda +\lambda /2+{L}_{x}^{k}({p}^{* },{q}^{* })\\ {y}^{* }={q}^{* }\cdot \lambda +\lambda /2+{L}_{y}^{k}({p}^{* },{q}^{* })\end{array}$$

with overall stride *λ*. If there are multiple keypoints *k* present then one can naturally take the local maxima of *S*^*k*^ to obtain the corresponding detections. Local maxima are identified via nonmaximum suppression with 2D max pooling of the score maps.

Thus, one obtains putative keypoint proposals from the score maps and location refinement fields. We then use the PAFs to assign the cost for linking two keypoints (within a putative animal). For any pair of keypoint proposals (that are connected via a limb as defined by the part affinity graph) we evaluate the affinity cost by integrating along line *γ* connecting two proposals, normalized by the length of *γ*:

2 $$\int \parallel {P}_{x,y}^{l}\parallel {\mathrm{d}}\gamma /\int {\mathrm{d}}\gamma$$

This integral is computed by sampling. Thus, for a given part affinity graph, one gets a (possibly) large number of detections and costs. The next step is to assemble those detections into animals.

### Data-driven PAF graph selection

To relieve the user from manually defining connections between keypoints, we developed an entirely data-driven procedure. Models are trained on a complete graph to learn all possible body part connections. We tested whether randomly pruning the complete marmoset skeleton (to 25, 50 and 75% of its original size: that is, 26, 52, 78 edges or 52, 104, 156 PAFs) to alleviate memory demands could still yield acceptable results. We found that pruning a large graph before training to a fourth of its original size was harmful (mAP loss of 15–20 points; Extended Data Fig. 9); at half and 75% of its size, a performance equivalent to that of the full graph was reached at 24 edges, although it remained about 1.5 mAP point under the maximal mAP score observed overall. Consequently, for large skeletons, a random subgraph is expected to yield only slightly inferior performance at a lesser computational cost.

The graph is then pruned based on edge discriminability power on the training set. For this purpose, within- and between-animal part affinity cost distributions (bin width 0.01) are evaluated (see Extended Data Fig. 4 for the mouse dataset). Edges are then ranked in decreasing order of their ability to separate both distributions—evaluated from the auROC curve. The smallest, data-driven graph is taken as the maximum spanning tree (that is, a subgraph covering all keypoints with the minimum possible number of edges that also maximizes part affinity costs). For graph search following a network’s evaluation, up to nine increasingly redundant graphs are formed by extending the minimal skeleton progressively with strongly discriminating edges in the order determined above. By contrast, baseline graphs are grown from a skeleton a user would naively draw, with edges iteratively added in reversed order (that is, from least to most discriminative). The graph jointly maximizing purity and the fraction of connected keypoints is the one retained to carry out the animal assemblies.

### Animal assembly

Animal assembly refers to the problem of assigning keypoints to individuals. Yet, reconstructing the full pose of multiple individuals from a set of detections is NP hard, as it amounts to solving a *k*-dimensional matching problem (a generalization of bipartite matching from 2 to *k* disjoint subsets)^9,38^. To make the task more tractable, we break the problem down into smaller matching tasks, in a manner akin to Cao et al.^9^.

For each edge type in the data-driven graph defined earlier, we first pick strong connections based on affinity costs alone. Following the identification of all optimal pairs of keypoints, we seek unambiguous individuals by searching this set of pairs for connected components—in graph theory, these are subsets of keypoints all reachable from one another but that do not share connection with any additional keypoint; consequently, only connectivity, but not spatial information, is taken into account. Breadth-first search runs in linear time complexity, which thus allows the rapid predetermination of unique individuals. Note that, unlike ref. ^9^, redundant connections are seamlessly handled and do not require changes in the formulation of the animal assembly. Then, remaining connections are sorted in descending order of their affinity costs (equation (2)) and greedily linked.

To further improve the assembly’s robustness to ambiguous connections (that is, a connection attempting to either link keypoints belonging to two distinct individuals or overwrite existing ones), the assembly procedure can be calibrated by determining the prior probability of an animal’s pose as a multivariate normal distribution over the distances between all pairs of keypoints. Mean and covariance are estimated from the labeled data via density estimation with Gaussian kernel and bandwidth automatically chosen according to Scott’s Rule. A skeleton is then only grown if the candidate connection reduces the Mahalanobis distance between the resulting configuration and the prior (referred to as with calibration in Fig. 2c). Last, our assembly’s implementation is fully parallelized to benefit greatly from multiple processors (Extended Data Fig. 5).

Optionally (and only when analyzing videos), affinity costs between body parts can be weighted so as to prioritize strong connections that were preferentially selected in the past frames. To this end, and inspired by ref. ^39^, we compute a temporal coherence cost as follows: $\frac{1}{j}\mathop{\sum }\nolimits_{i = 1}^{j}{e}^{-\gamma {{\Delta }}t{\left\Vert c-{c}_{n}\right\Vert }^{2}}$, where *γ* controls the influence of distant frames (and is set to 0.01 by default), *c* and *c*_*n*_ are the current connection and its closest neighbor in the relevant past frame and Δ*t* is the temporal gap separating these frames.

### Top-down pose estimation

In general, top-down pose estimation is characterized by two stages that require an object detector and a single animal pose estimation model^7^. This pipeline requires bounding box annotations (which can come from many different algorithms). Here, bounding boxes were determined from ground truth keypoint coordinates. If a box’s aspect ratio was lower than 4:1, its smallest side was extended by 10 pixels. Box bounds were further enlarged by 25 pixels to make sure the bounding boxes covered an animal’s entire body. We pad the cropped images to a square and then resize them to the original size (400 × 400) to keep the aspect ratio constant. Second, we retrain a model (either with or without PAFs) on training images cropped by these bounding boxes. For inference, we retain the best prediction per bounding box, as decided by detection confidence for the model without PAFs and with highest assembly score for the model with PAF. Finally, for evaluation, we map coordinates of our final predictions back to the original images.

### Detection performance and evaluation

To compare the human annotations with the model predictions we used the Euclidean distance to the closest predicted keypoint (r.m.s.e.) calculated per keypoint. Depending on the context, this metric is shown for a specific keypoint, averaged over all keypoints or averaged over a set of train or test images (Fig. 2a and Extended Data Fig. 3). Nonetheless, unnormalized pixel errors may be difficult to interpret in certain scenarios; for example, marmosets dramatically vary in size as they leap from the top to the bottom of the cage. Thus, we also calculated the percentage of correct keypoints (PCK) metric^21,40^; that is, the fraction of predicted keypoints that fall within a threshold distance from the location of the ground truth detection. PCK was computed in relation to a third of the tip–gill distance for the fish dataset, and a third of the left-right ear distance for the remaining ones.

Animal assembly quality was evaluated in terms of mAP computed over object keypoint similarity thresholds ranging from 0.50 to 0.95 in steps of 0.05, as is standard in human pose literature and COCO challenges^18^. Keypoint standard deviation was set to 0.1. As interpretable metrics, we also computed the number of ground truth keypoints left unconnected (after assembly) and purity—an additional criterion for quality that can be understood as the accuracy of the assignment of all keypoints of a putative subset to the most frequent ground truth animal identity within that subset^41^. Since pups are very hard to label consistently (see Extended Data Fig. 7 for examples), we allow flips between symmetric pairs of keypoints (end1 versus end2 or interm1 versus interm3, Extended Data Fig. 1) to be acceptable detection errors when evaluating keypoint similarity.

### Statistics for assessing data-driven method

Two-way, repeated-measures ANOVA were performed using Pinetwork flow minimizationngouin (v.0.5.0)^42^ to test whether graph size and assembling method (naive versus data-driven versus calibrated assembly) had an impact on the fraction of unconnected body parts and assembly purity. Since sphericity was violated, the Greenhouse–Geisser correction was applied. Provided a main effect was found, we conducted multiple post hoc (paired, two-sided) tests adjusted with Benjamini–Hochberg false discovery rate correction to locate pairwise differences. The Hedges’ *g* was calculated to report effect sizes between sets of observations.

### Comparison to state-of-the-art pose estimation models

For benchmarking, we compared our architectures to current state-of-the-art architectures on COCO^18^, a challenging, large-scale multi-human pose estimation benchmark. Specifically, we considered HRNet^11,43^ as well as ResNet backbones^22^ with Associative Embedding^10^ as implemented in the MMPose toolbox (https://github.com/open-mmlab/mmpose). We chose them as control group for their simplicity (ResNet) and performance (HRNet). We used the bottom-up variants of both models. The bottom-up variants leverage associative embedding as the grouping algorithms^10^. In particular, the bottom-up variant of HRNet we used has mAP that is comparable to the state-of-the-art model HigherHRNet^11^ in COCO (69.8 versus 70.6) for a multiple scale test and (65.4 versus 67.7) for a single scale test.

To fairly compare, we used the same train and test split. The total training epochs are set such that models from two groups see roughly same number of images. The hyperparameters search was manually performed to find the optimal hyperparameters. For a small dataset such as the tri-mouse and (largest) marmoset, we found that the default settings for excellent performance on COCO gave optimal accuracy except that we needed to modify the total training steps to match DeepLabCut’s. For both the marmoset and tri-mouse datasets, the initial learning rate was 0.0015. For the three mouse dataset, the total epochs is 3,000 epochs and the learning rate decayed by a factor of 10 at 600 and 1,000 epochs. For the marmoset dataset, we trained for 50 epochs and the learning rate decayed after 20 and 40 epochs. The batch size was 32 and 16 for ResNet-AE and HRNet-AE, respectively. For smaller datasets such as tri-mouse, fish and parenting, we found that a smaller learning rate and a smaller batch size gave better results; a total of 3,000 epochs were used. After hyper-parameter search, we set batch size to four and initial learning rate a 0.0001, which then decayed at 1,000 and 2,000 epochs. As within DeepLabCut, multiple scale test and flip test were not performed (which is, however, common for COCO evaluation). For the parenting dataset, MMPose models can only be trained on one dataset (simultaneously), which is why these models are not trained to predict the mouse, and we only compare the performance on the pups. Full results are shown in Fig. 2.

### Benchmarking idtacker.ai

We used version idtracker.ai^27^ v.3, taken from commit 6b89601b; we tested it on tri-mouse and marmoset data. We report the MOTA results in Extended Data Fig. 8. For marmoset data, reasonable parameters to segment individual animals with the GUI could not be found (likely due to the complex background), thus we performed a grid search for the valid minimum intensity threshold and maximum intensity threshold, the two critical parameters, by step 2 from range 0 to 255. Even with these efforts, we still could not get reasonable results (Supplementary Video 5); that is, MOTA was negative.

### DeepLabCut Tracking modules

Having seen that DeepLabCut provides a strong predictor for individuals and their keypoints, detections are linked across frames using a tracking-by-detection approach (for example, ref. ^44^). Thereby, we follow a divide-and-conquer strategy for (local) tracklet generation and tracklet stitching (Extended Data Fig. 4b,c). Specifically, we build on the Simple Online and Realtime Tracking framework (SORT^25^) to generate tracklets. The inter-frame displacement of assembled individuals is estimated via Kalman filter-based trackers. The task of associating these location estimates to the model detections is then formulated as a bipartite graph matching problem solved with the Hungarian algorithm, therefore guaranteeing a one-to-one correspondence across adjacent frames. Note that the trackers are agnostic to the type of skeleton (animal body plan), which render them robust and computationally efficient.

### Box tracker

Bounding boxes are a common and well-established representation for object tracking. Here they are computed from the keypoint coordinates of each assembled individual, and expanded by a margin optionally set by the user. The state *s* of an individual is parametrized as $s=[x,y,A,r,\dot{x},\dot{y},\dot{A}]$, where *x* and *y* are the 2D coordinates of the center of the bounding box; *A*, its area and *r*, its aspect ratio, together with their first time derivatives. Association between detected animals and tracker hypotheses is based on the intersection-over-union measure of overlap.

### Ellipse tracker

A 2*σ* covariance error ellipse is fitted to an individual’s detected keypoints. The state is modeled as $s=[x,y,h,w,\theta ,\dot{x},\dot{y},\dot{h},\dot{w},\dot{\theta }]$, where *x* and *y* are the 2D coordinates of the center of the ellipse; *h* and *w*, the lengths of its semi-axes and *θ*, its inclination relative to the horizontal. We anticipated that this parametrization would better capture subtle changes in body conformation, most apparent through changes in ellipse width and height and orientation. Moreover, an error ellipse confers robustness to outlier keypoints (for example, a prediction assigned to the wrong individual, which would cause the erroneous delineation of an animal’s boundary under the above-mentioned box tracking). In place of the ellipse overlap, the similarity cost *c* between detected and predicted ellipses is efficiently computed as: $c=0.8(1-d)+0.2(1-d)(\cos ({\theta }_{d}-{\theta }_{p}))$, where *d* is the Euclidean distance separating the ellipse centroids normalized by the length of the longest semi-axis.

The existence of untracked individuals in the scene is signaled by assembled detections with a similarity cost lower than iou_threshold (set to 0.6 in our experiments). In other words, the higher the similarity threshold, the more conservative and accurate the frame-by-frame assignment, at the expense of shorter and more numerous tracklets. On creation, a tracker is initialized with the required parameters described above, and all (unobservable) velocities are set to 0. To avoid tracking sporadic, spurious detections, a tracker is required to live for a minimum of min_hits consecutive frames, or is otherwise deleted. Occlusions and reidentification of individuals are handled with the free parameter max_age—the maximal number of consecutive frames tracks can remain undetected before the tracker is considered lost. We set both to 1 to delegate the tasks of tracklet reidentification and false positive filtering to our TrackletStitcher, as we shall see below.

### Tracklet stitching

Greedily linking individuals across frames is locally, but not globally, optimal. An elegant and efficient approach to reconstructing full trajectories (or tracks) from sparse tracklets is to cast the stitching task as a network flow minimization problem^45,46^. Each fully reconstructed track is equivalent to finding a flow through the graph from a source to a sink, subject to capacity constraints and whose overall linking cost is minimal (Extended Data Fig. 4c).

### Formulation

The tracklets collected after animal tracking are denoted as $\{{{{{\mathcal{T}}}}}_{1},...,{{{{\mathcal{T}}}}}_{n}\}$, and each contains a (temporally) ordered sequence of observations and time indices. Thereby, the observations are given as vectors of body part coordinates in pixels and likelihoods. In contrast to most approaches described in the literature, the proposed approach requires solely spatial and temporal information natively, while leveraging visual information (for example, animals’ identities predicted beforehand) is optional (see Fig. 3e for marmosets). This way, tracklet stitching is agnostic to the framework poses were estimated with, and works readily on previously collected kinematic data.

We construct a directed acyclic graph *G* = (*V*,*E*) using NetworkX^47^ to describe the affinity between multiple tracklets, where the *i*th node *V*_*i*_ corresponds to the *i*th tracklet ${{{{\mathcal{T}}}}}_{i}$, and *E* is the set of edges encoding the cost entailed by linking the two corresponding tracklets (or, in other words, the likelihood that they belong to the same track). In our experiments, tracklets shorter than five frames were flagged as residuals: they do not contribute to the construction of the graph and are incorporated only after stitching. This minimal tracklet length can be changed by a user. To drastically reduce the number of possible associations and make our approach scale efficiently to large videos, edge construction is limited to those tracklets that do not overlap in time (since an animal cannot occupy multiple spatial locations at any one instant) and temporally separated by no more than a certain number of frames. By default, this threshold is automatically taken as 1.5 × *τ*, where *τ* is the smallest temporal gap guaranteeing that all pairs of consecutive tracklets are connected. Alternatively, the maximal gap to consider can be programmatically specified. The source and the sink are two auxiliary nodes that supply and demand an amount of flow *k* equal to the number of tracks to form. Each node is virtually split in half: an input with unit demand and an output with unit supply, connected by a weightless edge. All other edges have unit capacity and a weight *w* calculated from the affinity models described in the next subsection. Altogether, these constraints ensure that all nodes are visited exactly once, which thus amounts to a problem similar to covering *G* with *k* node-disjoint paths at the lowest cost. We considered different affinities for linking tracklets (Fig. 4d).

### Affinity models

#### Motion affinity

Let us consider two nonoverlapping tracklets ${{{{\mathcal{T}}}}}_{1}$ and ${{{{\mathcal{T}}}}}_{2}$ consecutive in time. Their motion affinity is measured from the error between the true locations of their centroids (that is, unweighted average keypoint) and predictions made from their linear velocities. Specifically, we calculate a tracklet’s tail and head velocities by averaging instantaneous velocities over its three first and last data points (Fig. 4d). Assuming uniform, rectilinear motion, the centroid location of ${{{{\mathcal{T}}}}}_{1}$ at the starting frame of ${{{{\mathcal{T}}}}}_{2}$ is estimated, and we note *d*_f_ the distance between the forward prediction and the actual centroid coordinates. The same procedure is repeated backward in time, predicting the centroid location of ${{{{\mathcal{T}}}}}_{2}$ at the last frame of ${{{{\mathcal{T}}}}}_{1}$ knowing its tail velocity, yielding *d*_b_. Motion affinity is then taken as the average error distance.

#### Spatial proximity

If a pair of tracklets overlaps in time, we calculate the Euclidean distance between their centroids averaged over their overlapping portion. Otherwise, we evaluate the distance between a tracklet’s tail and the other’s head.

#### Shape similarity

Shape similarity between two tracklets is taken as the undirected Hausdorff distance between the two sets of keypoints. Although this measure provides only a crude approximation of the mismatch between two animals’ skeletons, it is defined for finite sets of points of unequal size; for example, it advantageously allows the comparison of skeletons with a different number of visible keypoints.

#### Dynamic similarity

To further disambiguate tracklets in the rare event that they are spatially and temporally close, and similar in shape, we propose to use motion dynamics in a manner akin to ref. ^48^. The procedure is fully data-driven, and requires no a priori knowledge of the animals’ behavior. In the absence of noise, the rank of the Hankel matrix—a matrix constructed by stacking delayed measurements of a tracklet’s centroid—theoretically determines the dimension of state space models; that is, it is a proxy for the complexity of the underlying dynamics^49^. If two tracklets originate from the same dynamical system, a single, low-order regressor should suffice to approximate them both. On the other hand, tracklets belonging to different tracks would require a higher-order (that is, more complex) model to explain their spatial evolution^48^. Low rank approximation of a noisy matrix, however, is a complex problem, as the matrix then tends to be full rank (that is, all its singular values are nonzero). For computational efficiency, we approximate the rank of a large numbers of potentially long tracklets using singular value decomposition via interpolative decomposition. Optimal low rank was chosen as the rank after which eigenvalues drop by less than 1%.

### Problem solution for stitching

The optimal flow solution can be found using a min-cost flow algorithm. We use NetworkX’s capacity scaling variant of the successive shortest augmenting path algorithm, which requires polynomial time for the assignment problem (that is, when all nodes have unit demands and supplies, ref. ^50^). Residual tracklets are then greedily added back to the newly stitched tracks at locations that guarantee time continuity and, when there are multiple candidates, minimize the distances to the neighboring tracklets. Note that although residuals are typically very short, making the assignment decisions error-prone. To improve robustness and simultaneously reduce complexity, association hypotheses between temporally close residual tracklets are stored in the form of small directed acyclic graphs during a preliminary forward screening pass. An hypothesis likelihood is then scored based on pairwise tracklet spatial overlap, and weighted longest paths are ultimately kept to locally grow longer, more confident residuals.

This tracklet stitching process is implemented in DeepLabCut and automatically carried out after assembly and tracking. The tracks can then also be manually refined in a dedicated GUI (Extended Data Fig. 1).

### Transformer for unsupervised ID tracking

To track unidentified animals we turn to metric learning^4^ with transformers, which are state-of-the-art for reID of humans and vehicles^51^. However, in contrast to ref. ^51^, we created a tracking approach and wanted to make use of the task-trained CNNs, and thus require fewer training data.

Specifically, we used the predicted coordinates of each tracklet (individual with temporal continuality) and extract features of 2,048 dimensions from the last layer of our (multi-task-trained) backbone network to form so called ‘keypoint embedding’, which contains embedding of each detected keypoint for every individual (and encode high-level visual features around the keypoint). Then we feed this keypoint embedding to a transformer that processes these embeddings and aggregates information globally. The transformer layers have four heads and four blocks with dimension of 768 and residual connections between blocks. The output of transformer layers are then followed by a multi-layer perceptron that outputs a vector of dimension 128 (more layers, as in ref. ^51^, actually gave a worse performance). We then use the output of the multi-layer perceptron to minimize triplet loss where we treat within tracklet embedding as anchor-positive pairs while tracklets from different individuals as anchor-negative pairs. For each test video, we extracted 10,000 triplets from the local-tracking approach (ellipse, to evaluate the capacity based on tracklets) and from the ground truth data (to evaluate the capacity of the approach; as triplets from ground truth tracks already are split into the correct number of animals). We then trained the transformer on 90% of the triplets, and evaluated it on the rest (Fig. 4). Thus, the transformer learns to recognize identities of each tracklet and we then use the cosine similarity as an additional cost to our graph. For this purpose, we used the transformer to extract 128 dimensional feature vectors (appearance embeddings) per keypoint embedding, which we then used for tracking (below).

### Tracking performance evaluation

Tracking performance was assessed with the field standard MOTA metrics^52^. Namely, we used https://github.com/cheind/py-motmetrics to compute MOTA, which evaluates a tracker’s overall performance at detecting and tracking individuals (all possible sources of errors considered: number of misses, of false positives and of mismatches (switches) respectively) independently of its ability to predict an individual’s location. MOTA is thereby the sum of three errors: the ratio of misses in the sequence, computed over the total number of objects present in all frames, the ratio of false positives and the ratio of mismatches^52^. The number of misses counts actual detections for which there are no matching trackers. The number of fragments indicates the number of times tracking was interrupted. The number of switches, occurring most often when two animals pass very close to one another or if tracking resumes with a different ID after an occlusion. In our software, remaining ID swaps are automatically flagged in the Refine Tracklets GUI (Extended Data Fig. 1) by identifying instants at which the *x* and *y* coordinates of a pair of keypoints simultaneously intersect each other^53^.

### Reporting Summary

Further information on research design is available in the Nature Research Reporting Summary linked to this article.

## Online content

Any methods, additional references, Nature Research reporting summaries, source data, extended data, supplementary information, acknowledgements, peer review information; details of author contributions and competing interests; and statements of data and code availability are available at 10.1038/s41592-022-01443-0.

## Supplementary information

Supplementary Information Supplementary Note 1 and Tables 1–8.

Reporting Summary

Supplementary Video 1 Predictions with DLCRNet MS5_ss-s4 for tri-mouse video clip.

Supplementary Video 2 Predictions with DLCRNet MS5_ss-s4 for parenting video clip.

Supplementary Video 3 Predictions with DLCRNet MS5_ss-s4 for marmoset video clip.

Supplementary Video 4 Predictions with DLCRNet MS5_ss-s4 for fish video clip.

Supplementary Video 5 Predictions with idtracker.ai on tri-mouse and marmoset data.

## Data Availability

For this study, we established four differently challenging multi-animal datasets from ecology and neuroscience. Data collection was institutionally approved: tri-mouse, parenting behavior, fish schooling from Harvard University IACUC and marmosets from MIT and Broad Institute IACUC. They are available to download, minus a small amount (30%) held out as benchmark competition data, at https://benchmark.deeplabcut.org/, and on Zenodo^54–57^. Findings in this paper can be replicated using the downloadable data and supplied code.

## Code availability

Code to use this package is found at https://github.com/DeepLabCut/DeepLabCut with a LGPL-3.0 License. Code to reproduce the figures from this work is found at https://github.com/DeepLabCut/maDLC_NatureMethods2022.
