# Supplementary material for: Multi-animal pose estimation, identification and tracking with DeepLabCut
Source: Nat Methods. 2022 Apr 12;19(4):496–504. doi: 10.1038/s41592-022-01443-0 (PMC9007739; doi:10.1038/s41592-022-01443-0)
Supplement: Supplementary file 1 — Supplementary Note 1 and Tables 1–8. [file 41592_2022_1443_MOESM1_ESM.pdf]

nature portfolio

https://doi.org/10.1038/s41592-022-01443-0

Supplementary information

Multi-animal pose estimation, identification and tracking with DeepLabCut

In the format provided by the authors and unedited

Supplementary Note 1 - Workflow and Usability of maDLC

In this work we have detailed various new algorithms for solving multi-animal pose estimation. Those tools are available in the DeepLabCut GitHub repository and the general workflow was expanded to accommodate multi-animal pose estimation projects with new GUIs for labeling, refining multi-animal pose, and refining tracklets. (**Extended Data 1**). The work presented in this paper is termed “maDeepLabCut” (maDLC) and is integrated into versions 2.2+ code base on GitHub, https://github.com/DeepLabCut/DeepLabCut, and the Python Package Index (PyPi). We provide Google Colab Notebooks, full project management software, graphic user interfaces, and Docker tooling to run this workflow on cloud and local computing resources. Note, this can also be run near real-time, as we have shown, assembly is fast (**Extended Data 5**) and the (local) tracking algorithm is an online method, which should allow for real-time processing. Lastly, we provide full user documentation at https://deeplabcut.github.io/DeepLabCut.

Supplementary Tables

Supplementary Table 1: Related to Figure 2: Graph Assembly Statistics. Graph size × assembling method interactions were found to have significant effects on purity in the tri-mouse and marmoset datasets, with small to medium effect sizes. In **Figure 2:**, the colored dots mark statistically significant interactions between graph size and assembling methods, as identified via two-way, repeated-measures ANOVAs. Red dots indicate a significant difference between baseline and data-driven, and blue dots, between data-driven and calibrated assemblies. Light red vertical bars highlight the graph automatically selected to balance the number of body parts left out after assembly and assembly purity.

Dataset	F-value	P-value	η_p^2
tri-mouse	16.309	<0.001	0.093
parenting	0.998	0.350	0.007
marmosets	21.108	<0.001	0.034
fish	2.660	0.061	0.084

Supplementary Table 2: Related to Figure 2: Fraction of unconnected keypoints. Two-way, repeated-measures ANOVAs revealed significant graph size × assembling method interactions in all but the tri-mouse dataset, with small to medium effect sizes.

Dataset	F-value	P-value	η_p^2
tri-mouse	6.590	0.011	0.040
parenting	3.387	0.039	0.022
marmosets	2.956	0.035	0.005
fish	3.923	0.017	0.119

Supplementary Table 3: Related to Figure 2: Statistics of post-hoc pairwise, two-sided, t-tests for differences in purity. A: first measurement; B: second measurement; p-corr: corrected p-values with Benjamini–Hochberg false discovery rate correction.

dataset	# graph	A	B	T-statistic	p-corr
tri-mouse	1	best	baseline	5.113	0.000
tri-mouse	2	best	baseline	5.300	0.000
tri-mouse	3	best	baseline	4.877	0.000
tri-mouse	4	best	baseline	5.192	0.000
tri-mouse	5	best	baseline	3.712	0.001
tri-mouse	6	best	baseline	3.078	0.004
tri-mouse	7	best	baseline	3.051	0.004
tri-mouse	8	best	baseline	2.843	0.008
tri-mouse	9	best	baseline	2.656	0.013
tri-mouse	10	best	calib	-3.559	0.001
marmosets	1	best	calib	6.163	0.000
marmosets	2	best	calib	4.242	0.000
marmosets	2	best	baseline	3.511	0.001
marmosets	3	best	baseline	6.195	0.000
marmosets	4	best	baseline	5.214	0.000
marmosets	5	best	baseline	4.387	0.000
marmosets	6	best	baseline	4.394	0.000
marmosets	7	best	baseline	4.286	0.000
marmosets	8	best	baseline	2.874	0.008
fish	1	best	calib	-4.243	0.002
fish	2	best	calib	-3.477	0.005
fish	3	best	baseline	3.339	0.006
fish	4	best	calib	-2.591	0.022
fish	4	best	baseline	3.143	0.009
fish	5	best	calib	-2.816	0.015
fish	6	best	calib	-3.713	0.003
fish	7	best	calib	-2.901	0.013

Supplementary Table 4: Related to Figure 2: Statistics of post-hoc pairwise, two-sided, t-tests for differences in unconnected keypoints. A: first measurement; B: second measurement; p-corr: corrected p-values with Benjamini–Hochberg false discovery rate correction.

dataset	# graph	A	B	T-statistic	p-corr
tri-mouse	1	best	calib	-2.913	0.012
tri-mouse	2	best	calib	-2.559	0.014
tri-mouse	3	best	calib	-2.891	0.012
tri-mouse	4	best	calib	-2.697	0.012
tri-mouse	5	best	calib	-2.697	0.012
marmosets	1	best	calib	-6.030	0.000
marmosets	2	best	calib	-5.759	0.000
marmosets	2	best	baseline	-2.490	0.018
marmosets	3	best	calib	-5.229	0.000
marmosets	3	best	baseline	-3.130	0.003
marmosets	4	best	calib	-4.969	0.000
marmosets	4	best	baseline	-3.023	0.004
marmosets	5	best	calib	-5.170	0.000
marmosets	5	best	baseline	-2.791	0.008
marmosets	6	best	calib	-5.021	0.000
marmosets	6	best	baseline	-2.421	0.021
marmosets	7	best	calib	-4.926	0.000
marmosets	7	best	baseline	-2.051	0.049
marmosets	8	best	calib	-5.180	0.000
marmosets	9	best	calib	-5.400	0.000
marmosets	10	best	calib	-5.506	0.000
fish	1	best	calib	-5.058	0.000
fish	2	best	calib	-5.434	0.000
fish	3	best	calib	-4.265	0.001
fish	4	best	calib	-4.310	0.001
fish	4	best	baseline	-2.727	0.021
fish	5	best	calib	-3.691	0.002
fish	7	best	calib	-3.834	0.002

Supplementary Table 5: Mean Average Precision (mAP) as a function of graph size for the **tri-mouse** dataset. 70/30 data split; test mAP only shown.

network name	G	11	17	23	29	35	41	47	53	59	66
EfficientNet-B7_s4	d	0.93	0.93	0.92	0.93	0.93	0.92	0.92	0.92	0.92	0.92
DLCRNet_ms4	d	0.92	0.92	0.92	0.92	0.92	0.92	0.92	0.92	0.91	0.91
EfficientNet-B7_s4	c	0.93	0.93	0.92	0.93	0.92	0.93	0.93	0.93	0.93	0.92
EfficientNet-B7	d	0.93	0.93	0.93	0.93	0.92	0.92	0.92	0.92	0.90	0.90
ResNet50_s4	d	0.93	0.92	0.92	0.93	0.92	0.92	0.92	0.92	0.91	0.91
ResNet50_s4	c	0.92	0.92	0.92	0.92	0.92	0.92	0.92	0.92	0.92	0.92

G = Graph: c = calibrated; d = data-driven; b = baseline; s = stride; MS = multistage; 30K iters; batchsize=8.

Supplementary Table 6: Mean Average Precision (mAP) as a function of graph size for the **parenting** dataset. 70/30 data split; test mAP only shown.

network name	G	4	5	6	7	8	9	10
EfficientNet-B7_s4	c	0.63	0.64	0.63	0.63	0.63	0.63	0.63
EfficientNet-B7_s4	d	0.64	0.64	0.64	0.63	0.63	0.63	0.63
DLCRNet_ms4	d	0.64	0.63	0.63	0.63	0.62	0.60	0.60
EfficientNet-B7	d	0.62	0.63	0.63	0.63	0.63	0.62	0.62
EfficientNet-B7	c	0.62	0.62	0.62	0.62	0.63	0.62	0.62
EfficientNet-B7_s4	b	0.64	0.64	0.63	0.62	0.63	0.63	0.63

G = Graph: c = calibrated; d = data-driven; b = baseline; s = stride; MS = multistage; 30K iters; batchsize=8.

Supplementary Table 7: Mean Average Precision (mAP) as a function of graph size for the **marmoset** dataset. 70/30 data split; test mAP only shown.

network name	G	14	24	34	44	54	64	74	84	94	105
DLCRNet_ms	d	0.88	0.88	0.89	0.89	0.88	0.88	0.88	0.88	0.87	0.87
ResNet50	d	0.87	0.88	0.89	0.88	0.88	0.88	0.88	0.87	0.87	0.87
DLCRNet_ms4	d	0.86	0.87	0.88	0.88	0.88	0.87	0.87	0.87	0.87	0.87
ResNet50_s4	d	0.85	0.86	0.86	0.87	0.86	0.86	0.86	0.86	0.86	0.86
EfficientNet-B7_s4	d	0.88	0.89	0.89	0.89	0.89	0.89	0.88	0.88	0.88	0.87
EfficientNet-B7	d	0.87	0.88	0.89	0.89	0.88	0.88	0.88	0.87	0.87	0.86

G = Graph: c = calibrated; d = data-driven; b = baseline; s = stride; MS = multistage; 200K iters; batchsize=8.

Supplementary Table 8: Mean Average Precision (mAP) as a function of graph size for the **fish** dataset. 70/30 data split; test mAP only shown.

network name	G	4	5	6	7	8	9	10
DLCRNet_ms4	d	0.715	0.710	0.73 5	0.728	0.715	0.716	0.707
DLCRNet_ms4	b	0.692	0.666	0.663	0.664	0.688	0.689	0.707
EfficientNet-B7_s4	b	0.679	0.664	0.658	0.663	0.680	0.685	0.693
EfficientNet-B7_s4	d	0.694	0.710	0.733	0.721	0.706	0.702	0.693
EfficientNet-B7_s4	c	0.688	0.695	0.731	0.714	0.706	0.711	0.685
ResNet50_s4	d	0.699	0.702	0.711	0.710	0.715	0.703	0.685

G = Graph: c = calibrated; d = data-driven; b = baseline; s = stride; MS = multistage; 30K iters; batchsize=8.
