# Supplementary material for: Multi-animal pose estimation, identification and tracking with DeepLabCut
Source: Nat Methods. 2022 Apr 12;19(4):496–504. doi: 10.1038/s41592-022-01443-0 (PMC9007739; doi:10.1038/s41592-022-01443-0)
Supplement: Supplementary file 2 — Reporting Summary [file 41592_2022_1443_MOESM2_ESM.pdf]

nature portfolio

Corresponding author(s): Mackenzie Mathis

Last updated by author(s): 2022-Jan-14

Reporting Summary

Nature Portfolio wishes to improve the reproducibility of the work that we publish. This form provides structure for consistency and transparency in reporting. For further information on Nature Portfolio policies, see our Editorial Policies and the Editorial Policy Checklist.

Statistics

For all statistical analyses, confirm that the following items are present in the figure legend, table legend, main text, or Methods section.

n/a	Confirmed	
☐	☒	The exact sample size (*n*) for each experimental group/condition, given as a discrete number and unit of measurement
☐	☒	A statement on whether measurements were taken from distinct samples or whether the same sample was measured repeatedly
☐	☒	The statistical test(s) used AND whether they are one- or two-sided<br>*Only common tests should be described solely by name; describe more complex techniques in the Methods section.*
☐	☒	A description of all covariates tested
☐	☒	A description of any assumptions or corrections, such as tests of normality and adjustment for multiple comparisons
☐	☒	A full description of the statistical parameters including central tendency (e.g. means) or other basic estimates (e.g. regression coefficient) AND variation (e.g. standard deviation) or associated estimates of uncertainty (e.g. confidence intervals)
☐	☒	For null hypothesis testing, the test statistic (e.g. *F*, *t*, *r*) with confidence intervals, effect sizes, degrees of freedom and *P* value noted<br>*Give P values as exact values whenever suitable.*
☒	☐	For Bayesian analysis, information on the choice of priors and Markov chain Monte Carlo settings
☒	☐	For hierarchical and complex designs, identification of the appropriate level for tests and full reporting of outcomes
☒	☐	Estimates of effect sizes (e.g. Cohen's *d*, Pearson's *r*), indicating how they were calculated

Our web collection on statistics for biologists contains articles on many of the points above.

Software and code

Policy information about availability of computer code

Data collection	DeepLabCut versions 2.2 - 2.2.0.5 was used to label, analyze and make data splits. Code is at https://github.com/DeepLabCut/DeepLabCut. DeepLabCut's dependencies are listed here: https://github.com/DeepLabCut/DeepLabCut/blob/master/requirements.txt, and are as follows: ipython, filterpy, h5py, ruamel.yaml>=0.15.0, intel-openmp, imgaug==0.4.0, jupyter-book>=0.7.0b, numba, matplotlib, networkx, numpy, pandas>=1.0.1, patsy, pyyaml, setuptools, scikit-image>=0.17,<=0.18.1, scikit-learn, scipy>=1.4, six, statsmodels>=0.11, torch, tensorflow>=2.0, tables, tensorpack, tf_slim, tqdm, moviepy, Pillow>=7.1
Data analysis	DeepLabCut versions 2.2 - 2.2.0.5 was used to analyze the data. Code is at https://github.com/DeepLabCut/DeepLabCut. DeepLabCut's dependencies are listed here: https://github.com/DeepLabCut/DeepLabCut/blob/master/requirements.txt, and are as follows: ipython, filterpy, h5py, ruamel.yaml>=0.15.0, intel-openmp, imgaug==0.4.0, jupyter-book>=0.7.0b, numba, matplotlib, networkx, numpy, pandas>=1.0.1, patsy, pyyaml, setuptools, scikit-image>=0.17,<=0.18.1, scikit-learn, scipy>=1.4, six, statsmodels>=0.11, torch, tensorflow>=2.0, tables, tensorpack, tf_slim, tqdm, moviepy, Pillow>=7.1

For manuscripts utilizing custom algorithms or software that are central to the research but not yet described in published literature, software must be made available to editors and reviewers. We strongly encourage code deposition in a community repository (e.g. GitHub). See the Nature Portfolio guidelines for submitting code & software for further information.

Data

Policy information about availability of data

All manuscripts must include a data availability statement. This statement should provide the following information, where applicable:

- Accession codes, unique identifiers, or web links for publicly available datasets
- A description of any restrictions on data availability
- For clinical datasets or third party data, please ensure that the statement adheres to our policy

Data links can be found at benchmark.deeplabcut.org and on Zenodo: https://zenodo.org/record/5851157#.YeMNJVjMIcQ, https://zenodo.org/record/5851109#.YeMNMVjMIcQ, https://zenodo.org/record/5849371#.YeMNP1jMIcQ, https://zenodo.org/record/5849286#.YeMNS1jMIcQ.

Field-specific reporting

Please select the one below that is the best fit for your research. If you are not sure, read the appropriate sections before making your selection.

☒ Life sciences ☐ Behavioural & social sciences ☐ Ecological, evolutionary & environmental sciences

For a reference copy of the document with all sections, see nature.com/documents/nr-reporting-summary-flat.pdf

Life sciences study design

All studies must disclose on these points even when the disclosure is negative.

Sample size	Sample size was not predetermined, but validated in the paper and similar to Mathis et al, 2018 Nature Neuroscience.
Data exclusions	No data was excluded.
Replication	Yes, models and hyperparameters were validated and repeated.
Randomization	Data was randomly split into training and test sets.
Blinding	Authors were blinded to the randomization of the data at the initial split, and this was not modified. For downstream analysis the experimenters were not blinded (as this was not required at this point).

Reporting for specific materials, systems and methods

We require information from authors about some types of materials, experimental systems and methods used in many studies. Here, indicate whether each material, system or method listed is relevant to your study. If you are not sure if a list item applies to your research, read the appropriate section before selecting a response.

Materials & experimental systems

n/a	Involved in the study	
☒	☐	Antibodies
☒	☐	Eukaryotic cell lines
☒	☐	Palaeontology and archaeology
☐	☐	Animals and other organisms
☒	☐	Human research participants
☒	☐	Clinical data
☒	☐	Dual use research of concern

Methods

n/a	Involved in the study	
☒	☐	ChIP-seq
☒	☐	Flow cytometry
☒	☐	MRI-based neuroimaging

Animals and other organisms

Policy information about studies involving animals; ARRIVE guidelines recommended for reporting animal research

Laboratory animals	Tri-Mouse dataset: Three male wild-type (C57BL/6J) (temperature of housing was 20-25C, humidity 20-50%) were used.<br>Mouse Parenting dataset: adult female (age was less than 180 days old) female C57Bl6/J and two pups (4 days old, sex unknown) temperature of housing was 20-25C, humidity 20-50%.<br>Marmoset dataset: common marmosets (Callithrix jacchus) were used. Both sexes (pair housed) age ranged from 2-12 years old were used.<br>Fish dataset: inland silversides (Menidia beryllina), n=14 individuals per school, sex unknown but likely to be equal female/male, aged approximately 9 months.

Wild animals	No wild animals were used in this study.
Field-collected samples	No field-collected samples were used in this study.
Ethics oversight	Ethical approval for parenting, tri-mouse, fish were from Harvard University IACUC; marmosets were approved by the MIT and Broad IACUC.

Note that full information on the approval of the study protocol must also be provided in the manuscript.
